# Planned mode of delivery after previous cesarean section and short-term maternal and perinatal outcomes: A population-based record linkage cohort study in Scotland

**DOI:** 10.1371/journal.pmed.1002913

**Published:** 2019-09-24

**Authors:** Kathryn E. Fitzpatrick, Jennifer J. Kurinczuk, Sohinee Bhattacharya, Maria A. Quigley

**Affiliations:** 1 National Perinatal Epidemiology Unit, Nuffield Department of Population Health, University of Oxford, Oxford, United Kingdom; 2 The Institute of Applied Health Sciences, University of Aberdeen, Aberdeen, United Kingdom; Cambridge University, UNITED KINGDOM

## Abstract

**Background:**

Policy consensus in high-income countries supports offering pregnant women with previous cesarean section a choice between planning an elective repeat cesarean section (ERCS) or attempting a vaginal birth, known as a planned vaginal birth after previous cesarean (VBAC), provided they do not have contraindications to planned VBAC. However, robust comprehensive information on the associated outcomes to counsel eligible women about this choice is lacking. This study investigated the short-term maternal and perinatal outcomes associated with planned mode of delivery after previous cesarean section among women delivering a term singleton and considered eligible to have a planned VBAC.

**Methods and findings:**

A population-based cohort of 74,043 term singleton births in Scotland between 2002 and 2015 to women with one or more previous cesarean sections was conducted using linked Scottish national datasets. Logistic or modified Poisson regression, as appropriate, was used to estimate the effect of planned mode of delivery on maternal and perinatal outcomes adjusted for sociodemographic, maternal medical, and obstetric-related characteristics. A total of 45,579 women gave birth by ERCS, and 28,464 had a planned VBAC, 28.4% of whom went on to have an in-labor nonelective repeat cesarean section. Compared to women delivering by ERCS, those who had a planned VBAC were significantly more likely to have uterine rupture (0.24%, *n* = 69 versus 0.04%, *n* = 17, adjusted odds ratio [aOR] 7.3, 95% confidence interval [CI] 3.9–13.9, *p* < 0.001), a blood transfusion (1.14%, *n* = 324 versus 0.50%, *n* = 226, aOR 2.3, 95% CI 1.9–2.8, *p* < 0.001), puerperal sepsis (0.27%, *n* = 76 versus 0.17%, *n* = 78, aOR 1.8, 95% CI 1.3–2.7, *p* = 0.002), and surgical injury (0.17% versus 0.09%, *n* = 40, aOR 3.0, 95% CI 1.8–4.8, *p* < 0.001) and experience adverse perinatal outcomes including perinatal death, admission to a neonatal unit, resuscitation requiring drugs and/or intubation, and an Apgar score < 7 at 5 minutes (7.99%, *n* = 2,049 versus 6.37%, *n* = 2,570, aOR 1.6, 95% CI 1.5–1.7, *p* < 0.001). However, women who had a planned VBAC were more likely than those delivering by ERCS to breastfeed at birth or hospital discharge (63.6%, *n* = 14,906 versus 54.5%, *n* = 21,403, adjusted risk ratio [aRR] 1.2, 95% CI 1.1–1.2, *p* < 0.001) and were more likely to breastfeed at 6–8 weeks postpartum (43.6%, *n* = 10,496 versus 34.5%, *n* = 13,556, aRR 1.2, 95% CI 1.2–1.3, *p* < 0.001). The effect of planned mode of delivery on the mother’s risk of having a postnatal stay greater than 5 days, an overnight readmission to hospital within 42 days of birth, and other puerperal infection varied according to whether she had any prior vaginal deliveries and, in the case of length of postnatal stay, also varied according to the number of prior cesarean sections. The study is mainly limited by the potential for residual confounding and misclassification bias.

**Conclusions:**

Among women considered eligible to have a planned VBAC, planned VBAC compared to ERCS is associated with an increased risk of the mother having serious birth-related maternal and perinatal complications. Conversely, planned VBAC is associated with an increased likelihood of breastfeeding, whereas the effect on other maternal outcomes differs according to whether a woman has any prior vaginal deliveries and the number of prior cesarean sections she has had. However, the absolute risk of adverse outcomes is small for either delivery approach. This information can be used to counsel and manage the increasing number of women with previous cesarean section, but more research is needed on longer-term outcomes.

## Author summary

**Why was this study done?:**

- Many countries have seen a rise in their cesarean section rate, leading to an increasing proportion of women having a subsequent pregnancy with a history of previous cesarean section.
- Pregnant women who have had previous cesarean section may be offered a choice between planning an elective repeat cesarean section or attempting a vaginal birth, providing they do not have medical reasons why a planned cesarean is considered the safer or more advisable option.
- Clinical guidelines recommend that women should be counseled about the benefits and harms of planning to have another cesarean section compared to attempting a vaginal delivery to help with this decision. However, there is a lack of robust comprehensive information on the associated outcomes to counsel eligible women about this choice.

**What did the researchers do and find?:**

- We undertook a cohort study of 74,043 term singleton births in Scotland to women with one or more previous cesarean sections to determine the short-term maternal and perinatal outcomes associated with planned mode of delivery after previous cesarean section.
- We found that attempting a vaginal birth compared to having an elective repeat cesarean section is associated with an increased risk of the mother having serious birth-related complications such as uterine rupture and blood transfusion, as well as an increased risk of adverse perinatal outcomes.
- Conversely, we found that attempting a vaginal birth is associated with an increased likelihood of breastfeeding, whereas the association with other maternal outcomes appears to differ according to whether a woman has any prior vaginal deliveries and the number of previous cesarean sections she has had.
- However, the absolute risk of adverse maternal and perinatal outcomes was found to be small for either delivery approach: overall, just 1.8% of those attempting a vaginal birth and 0.8% of those having an elective repeat cesarean section experienced serious maternal complications, and 8.0% of those attempting a vaginal birth and 6.4% of those having an elective repeat cesarean section experienced one or more of the adverse perinatal outcomes considered.

**What do these findings mean?:**

- Our findings can be used to counsel and manage women with previous cesarean section and should be considered alongside existing evidence on the increased risk of serious maternal morbidity in subsequent pregnancies associated with elective repeat cesarean section.

## Introduction

Cesarean section is now one of the most common surgical procedures performed, with many parts of the world having seen a sharp rise in their cesarean section rates in recent years [1–4]. In the United Kingdom, nearly 30% of all births are now delivered by cesarean section [5–7]. The rise in cesarean section rates has led to an increasing proportion of women embarking on a subsequent pregnancy with a history of previous cesarean section. Broad policy consensus in high-income countries supports offering pregnant women who have had previous cesarean section a choice between planning to have another cesarean, known as an elective repeat cesarean section (ERCS), or attempting a vaginal birth, known as a planned vaginal birth after previous cesarean (VBAC), also known as a trial of labor after previous cesarean (TOLAC). This is provided that they do not have contraindications to planned VBAC such as placenta previa or transverse lie.

Current clinical guidelines [8–11] advise that pregnant women who have had previous cesarean section should be counseled about the risks and benefits of planned VBAC compared to ERCS to help them make an informed decision regarding this aspect of their maternity care. Furthermore, previous research suggests that although several factors may influence women’s decisions about planned mode of delivery after prior cesarean section, many would find it helpful to have access to accurate, comprehensive, and well-balanced information about the associated risks and benefits when making what is often viewed as a very difficult decision [12–15]. However, a number of significant limitations have been highlighted with the existing evidence [16]. These include a lack of comparability between the comparison groups; specifically, it is often unclear whether women included in the ERCS group were eligible to have a planned VBAC. Furthermore, although many studies have reported the rare risk of uterine rupture associated with planned VBAC and the risk of placental abnormalities in subsequent pregnancies associated with ERCS, contemporary, adequately powered, population-based studies comparing a wider range of outcomes for women and their children of planned VBAC with those of ERCS are limited. The aim of this population-based study was to investigate the short-term maternal and perinatal outcomes associated with planned mode of delivery after previous cesarean section among women delivering a singleton at term and considered eligible to have a planned VBAC.

## Methods

### Study design and data sources

A population-based retrospective cohort study was conducted by linking six routinely available Scottish national datasets:

1. National Records of Scotland (NRS) live births and stillbirths, which contains statutory data about all live births and stillbirths occurring in Scotland compiled from birth registrations and subject to various quality checks [17].
2. The Scottish Morbidity Record Maternity Inpatient and Day Case dataset (SMR02), which contains data on all inpatient and day case discharges from obstetric specialties in the National Health Service (NHS) Scotland, including information on maternal and infant characteristics, clinical management, and obstetric complications. Although not statutory, SMR02 has had a national coverage of around 98% of all births in NRS since the late 1970s, with some of the shortfall due to data about home births and at non-NHS hospitals not being available from SMR02 [18]. The data are subject to regular quality assurance checks [19].
3. The Scottish Morbidity Record General/Acute Inpatient and Day case dataset (SMR01), which contains demographic and clinical data on all hospital inpatient and day case discharges from acute specialties in NHS Scotland. The data are subject to regular quality assurance checks and are estimated to be 99% complete [20].
4. NRS deaths dataset, which contains statutory data about all deaths occurring in Scotland compiled from death registrations and subject to various quality checks [17].
5. The Scottish Stillbirth and Infant Death Survey (SSBID), which is a register that classified all stillbirths and neonatal deaths from 1985 to 2012 that were registered with NRS [21]. Classification of the cause of death was performed by a single medically qualified individual using clinical information from local coordinators and pathologists.
6. The Child Health Surveillance Programme Pre-School system (CHSP-PS), which contains data collected as part of child health reviews carried out on preschool children in Scotland, including infant feeding information. The number of NHS health boards in Scotland using CHSP-PS has increased over time, with the quality of information recorded on infant feeding reported to be high [22].

All linkages were undertaken by a third party under contract to the Information Services Division (ISD) Scotland using exact matching of the mother or child’s community health index (CHI) number, a unique person identifier used in Scotland. A complete list of the data sources, codes, and database fields used is provided in S1 Table.

### Study population

The study population included singleton births at term (37–41 completed weeks gestation) in Scotland, UK, between 1 January 2002 and 31 December 2015 to women with one or more previous cesarean sections. Births to women with one or more previous cesarean sections were identified in the SMR02 as delivery episodes that had a previous cesarean section field value of ≥1 or delivery episodes to women who had at least one previous delivery episode in which the mode of delivery was recorded as cesarean section and/or a cesarean section operation code was present, using SMR02 records going back as far as 1981.

Births to women not considered eligible to have a planned VBAC based on current UK guidelines [8,9] were excluded. This excluded births to women with any of the following: noncephalic presentation at delivery, placenta previa, abdominal pregnancy, known or suspected disproportion of maternal and/or fetal origin, tumor of corpus uteri, or birth by prelabor nonelective cesarean section. Births to women who had an antepartum stillbirth were also excluded, as vaginal birth is usually recommended in this situation [23]. Stillbirths missing time of death in relation to birth, births missing mode of delivery or gestational age at delivery, births delivered by nonelective cesarean section with missing information about duration of labor, and instances in which the number of previous cesarean sections was greater than a woman’s recorded parity were also excluded.

### Exposures

Among the study population of women with one or more previous cesarean sections, the primary exposure of interest was planned mode of delivery with planned VBAC (women delivering vaginally or by nonelective cesarean section with a duration of labor of 1 or more hours) compared to ERCS (women recorded as having an elective cesarean section). ISD Scotland defines a cesarean as elective if it is performed during the day with both the patient and staff fully prepared.

Analysis was also performed according to whether planned VBAC was attempted with or without labor induction (including surgical and/or medical induction) compared to ERCS. For comparative purposes, analysis was additionally conducted according to actual mode of delivery, defined as follows: women who were recorded as having a vaginal birth were classified as having a successful VBAC, women who were recorded as having a nonelective cesarean section with a duration of labor of 1 or more hours were classified as having an in-labor nonelective repeat cesarean section, and women who were recorded as having an elective cesarean section were classified as having an ERCS.

### Outcomes

The following predetermined maternal outcomes as defined in S1 Table were investigated: uterine rupture, blood transfusion, peripartum hysterectomy, puerperal sepsis, other puerperal infections, surgical injury (damage to bowel, bladder, or ureter requiring surgical repair), length of postnatal hospital stay, overnight readmission to hospital within 42 days of giving birth, any breastfeeding at birth or hospital discharge, exclusive breastfeeding, and any breastfeeding (exclusive or mixed breast and formula) at around 6–8 weeks after birth. The absolute risk of third- or fourth-degree perineal tears was also described among those who had a planned VBAC. Women who died before discharge or were not discharged within 42 days of birth were excluded from the analysis of maternal overnight readmission to hospital. Intrapartum stillbirths were excluded from the analysis of breastfeeding at birth or hospital discharge, and intrapartum stillbirths as well as neonatal deaths (deaths within 4 weeks of birth) were excluded from the analysis of breastfeeding at around 6–8 weeks after birth.

Predetermined perinatal outcomes investigated were intrapartum stillbirth or neonatal death, admission to a neonatal unit, resuscitation requiring drugs and/or intubation, and Apgar score < 7 at 5 minutes. A composite outcome comprising any of these adverse perinatal outcomes was also examined. Deaths due to congenital abnormalities were excluded from all perinatal outcomes, and all intrapartum stillbirths were excluded from the analysis of neonatal unit admission, resuscitation, and Apgar score.

### Statistical analysis

All analyses were prespecified as described in the methods section based on clear hypotheses and biological plausibility. We did not publish or preregister an analysis plan, but a summary of the proposed study exposures, outcomes, and statistical methods was included as part of the application to the Public Benefit and Privacy Panel for Health and Social Care Scotland to obtain the data (see S1 Text) and as part of the funding application to the National Institute for Health Research (NIHR). The characteristics of the primary exposure groups (planned VBAC and ERCS) were compared using descriptive statistics. Logistic regression was used to estimate odds ratios (ORs) and 95% confidence intervals (CIs) for rare binary outcomes (affecting <10% of population), and modified Poisson regression was used to estimate risk ratios (RRs) and 95% CIs for common binary outcomes, recognizing that ORs do not give a good approximation of the relative risk when the outcome is common [24]. All models were adjusted for year of delivery to account for any temporal changes (hereafter referred to as the “base model”). To examine the relative influence of sociodemographic, maternal medical, and obstetric-related factors on the association between the exposure variables and outcome in question, models were adjusted in a hierarchical fashion: model A was adjusted for a priori sociodemographic factors; model B was additionally adjusted for a priori maternal medical and pregnancy-related factors; models assessing breastfeeding outcomes and the risk of adverse perinatal outcomes were additionally adjusted for a priori infant-related factors (model C). The above adjustments were only performed when there was a minimum of 5–9 outcome events per coefficient in the model [25,26]. All models were determined a priori based on preexisting hypotheses or evidence on what factors are thought to potentially explain any association between the exposure and outcome in question [16,27–29].

Continuous variables were examined for evidence of departure from linearity in the models for each outcome using fractional polynomials, a method that involves fitting multiple power transformations of the continuous variable including the following powers: −2, −1, −0.5, 0, 1, 2, and 3 [30]. Continuous variables with insufficient evidence of nonlinearity were treated as continuous linear terms when adjusting for them in the analysis, whereas those showing evidence of nonlinearity were fitted using the best-fitting power transformation of the variable. Evidence of linearity was assessed using a *p*-value comparing the best-fitting fractional polynomial model to a linear model; *p*-values greater than or equal to 0.01 were interpreted as insufficient evidence of departure from linearity. Plausible effect modification between the primary exposure of interest and covariates (maternal age, number of previous cesarean sections, any prior vaginal delivery, interpregnancy interval, and maternal BMI) was tested in the full regression models by the addition of interaction terms to the model. Robust standard errors were used to account for the lack of independence in the data of women who had more than one eligible delivery in the study period. All *p*-values were two-sided with the significance level set at <0.05, except for interaction tests and tests of departure from linearity, in which the significance level was set at <0.01 to allow for multiple testing. All analyses were conducted in StataMP version 14 (Statacorp, College Station, TX, United States). The study is reported as per the REporting of studies Conducted using Observational Routinely-collected Data (RECORD) guideline (S1 RECORD Checklist).

### Missing data

A total of 38.6% of the study population had missing data on one or more covariates (see Table 1 footnotes for more details). The characteristics of those with missing data differed from those with complete data, suggesting the data were not missing completely at random (data available from author on request). Excluding those with missing data from the analysis (complete case analysis) would potentially introduce bias as well as reduce study power. Multiple imputation was therefore used to impute the following partially observed covariates: prior vaginal delivery, interpregnancy interval, maternal smoking status, maternal BMI, and birth weight centile. All covariates and the outcome of interest were included in the imputation models, and 40 imputations were performed on the basis of the suggested rule of thumb that the number of imputations should be at least equal to the percentage of incomplete cases [31]. When there appeared from the complete case analysis to be evidence of nonlinear covariate effects or interactions between covariates and the primary exposure of interest, a multiple imputation method using a recently developed extension to the chained equations approach was used [32]. This was to ensure missing covariate values were imputed from imputation models that were compatible with the analysis models. Otherwise, multiple imputation using the normal chained equations method was used.

**Table 1:** Characteristics of study cohort by planned mode of delivery after previous cesarean section.

	ERCS *n* (%)^a^, unless otherwise stated (*n* = 45,579)	Planned VBAC *n* (%)^a^, unless otherwise stated (*n* = 28,464)
**Sociodemographic characteristics**		
Maternal age (years)		
Less than 25	4,372 (9.6)	3,546 (12.5)
25–29	10,104 (22.2)	6,698 (23.5)
30–34	15,854 (34.8)	9,994 (35.1)
35–39	12,249 (26.9)	6,856 (24.1)
40 or more	3,000 (6.6)	1,370 (4.8)
Median (IQR) maternal age (years)	32 (28–36)	32 (28–35)
Mother’s country of birth		
UK	40,420 (88.7)	24,814 (87.2)
Non-UK	5,159 (11.3)	3,650 (12.8)
Marital status/registration type		
Married or joint registration/same address	40,786 (89.5)	25,290 (88.8)
Joint registration/different address	3,321 (7.3)	2,100 (7.4)
Sole registration	1,472 (3.2)	1,074 (3.8)
Socioeconomic status^b^		
Managerial/professional	21,951 (48.2)	13,230 (46.5)
Intermediate	10,115 (22.2)	5,988 (21.0)
Routine/manual	11,678 (25.6)	7,819 (27.5)
Other^c^	1,835 (4.0)	1,427 (5.0)
**Maternal medical and pregnancy-related characteristics**		
Number of previous cesarean sections		
1	33,956 (74.5)	27,509 (96.6)
2 or more	11,623 (25.5)	955 (3.4)
Median (IQR) number of previous cesarean sections	1 (1–2)	1 (1–1)
Any prior vaginal deliveryΨ		
No	37,523 (82.7)	17,380 (61.2)
Yes	7,874 (17.3)	10,997 (38.8)
Interpregnancy interval (months)Ψ		
24 or more	25,321 (60.0)	13,720 (56.4)
12–23	10,939 (25.9)	6,700 (27.5)
Less than 12	5,927 (14.0)	3,915 (16.1)
Median (IQR) interpregnancy interval (months)Ψ	29 (17–50)	27 (16–48)
Mother smoked at bookingΨ		
No	34,991 (83.3)	20,677 (78.2)
Yes	7,028 (16.7)	5,773 (21.8)
Maternal BMI at booking (kg/m^2^)Ψ		
Less than 25	11,957 (35.9)	8,741 (46.6)
25–29.9	10,181 (30.5)	5,738 (30.6)
30 or more	11,194 (33.6)	4,294 (22.9)
Median (IQR) BMI at booking (kg/m^2^)Ψ	27 (24–32)	25 (23–29)
Hypertensive disorder	2,258 (5.0)	1,634 (5.7)
Diabetes	1,960 (4.3)	479 (1.7)
Prelabor rupture of membranes	451 (1.0)	2,448 (8.6)
**Infant-related characteristics**		
Male infant	23,218 (50.9)	14,651 (51.5)
Gestational age at delivery (weeks)		
39–41	31,880 (69.9)	22,758 (80.0)
37–38	13,699 (30.1)	5,706 (20.0)
Median (IQR) gestational age at delivery (weeks)	39 (38–39)	40 (39–40)
Birth weight centile^d^		
10th–90th	35,930 (79.3)	22,928 (80.8)
Less than 10th	2,640 (5.8)	2,890 (10.2)
More than 90th	6,749 (14.9)	2,555 (9.0)

^a^Percentage of those with complete data.

^b^Socioeconomic status of mother for sole registered birth or highest of mother’s or father’s socioeconomic status for births registered inside marriage or jointly registered by both parents outside marriage. Socioeconomic status defined by NS-SEC based on occupation and employment status.

^c^Other includes never worked/long-term unemployed, student, not stated, or not classifiable.

^d^Derived from gestational age at delivery, birth weight, and gender of child using sex-specific birth weight for gestational age centiles as reported by Bonellie and colleagues [33].

ΨMissing data: any prior vaginal delivery 269 (0.36%); interpregnancy interval 7,521 (10.16%); maternal smoking status 5,574 (7.53%); maternal BMI 21,938 (29.63%); birth weight centile 351 (0.47%).

Abbreviations: ERCS, elective repeat cesarean section; IQR, interquartile range; NS-SEC, National Statistics Socio-Economic Classification; VBAC, vaginal birth after previous cesarean

### Sensitivity analyses

Three sensitivity analyses were conducted. First, a complete case analysis for each outcome studied was performed. Second, recognizing that the criterion used to define planned mode of delivery could misclassify some women who planned ERCS but went into labor before their scheduled delivery date, the analysis was confined to births delivered at 39 or more weeks of gestation. Since 2004, this is the gestation recommended by UK guidelines to carry out an ERCS [8,9]. Third, an analysis was conducted in which intrapartum stillbirths and neonatal deaths, excluding deaths from congenital abnormalities, were identified using SSBID rather than NRS deaths data. NRS deaths data were available for the whole study period, with cause of death coded in accordance with ICD-10. SSBID data were collected until 2012, and a revised classification system for cause of death was introduced in the last 2 years of the survey, so only data until 2010 were used for the sensitivity analysis. Up until 2010, the cause of stillbirth or neonatal death was classified using the Scottish Obstetric and Paediatric system last modified in 1987 [21].

### Approvals

The study did not require UK National Research Ethics Service (NRES) approval, as it involved the analysis of anonymized secondary data sources. However, approval for the study was obtained from the Public Benefit and Privacy Panel for Health and Social Care Scotland (application number 1516–0196).

## Results

**Fig 1:**
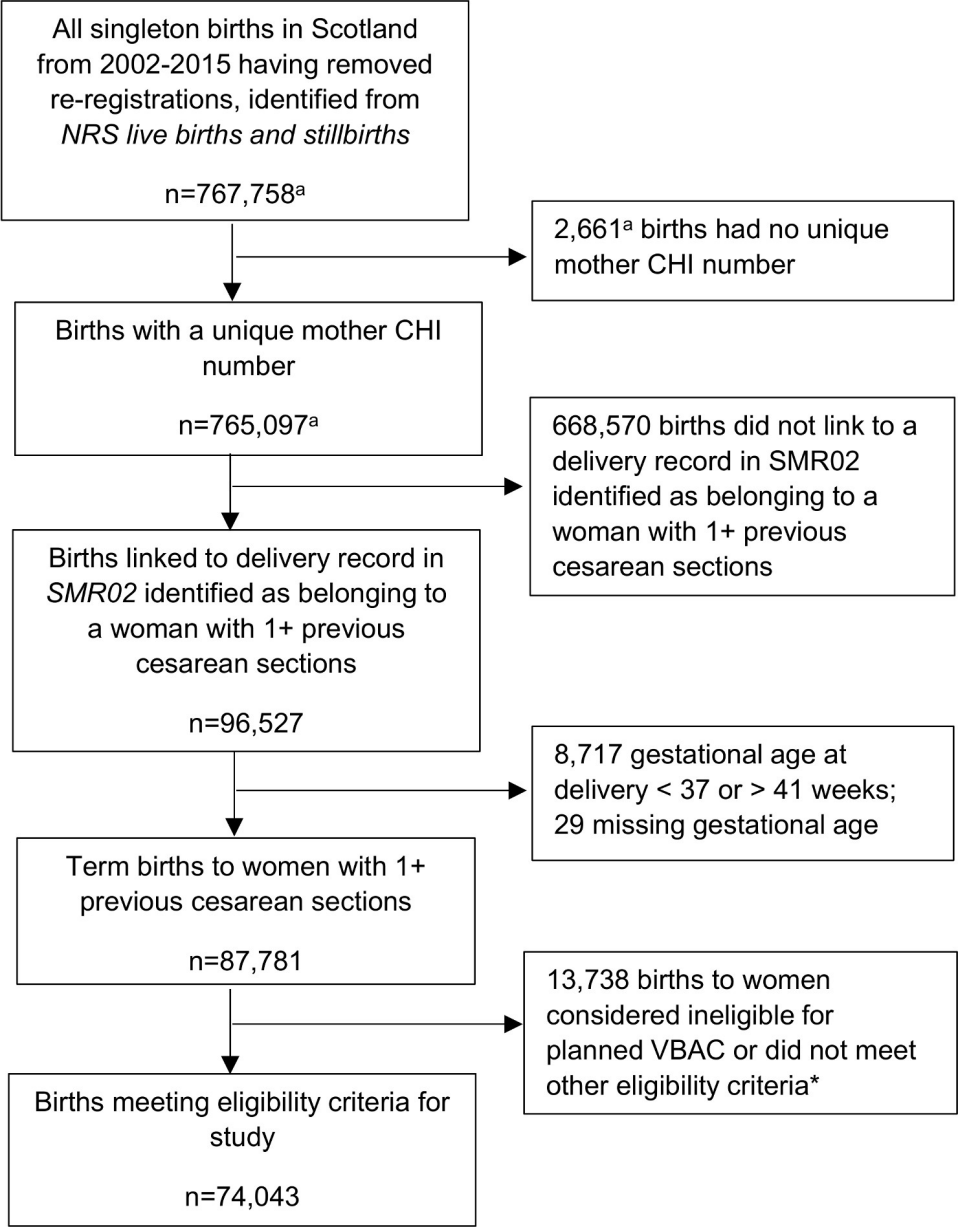
Flow diagram of cohort identification. *Ineligible for planned VBAC or did not meet other eligibility criteria for study because of one or more of the following: noncephalic presentation at delivery (*n* = 6,100), placenta previa (*n* = 570), abdominal pregnancy (*n* = 1), known or suspected disproportion of maternal and/or fetal origin (*n* = 113), tumor of corpus uteri (*n* = 268), birth by prelabor nonelective cesarean section (*n* = 5,954), antepartum stillbirth (*n* = 131), stillbirth missing time of death in relation to birth (*n* = 7), missing information on mode of delivery (*n* = 10); delivering by nonelective cesarean section missing information about duration of labor (*n* = 1,291), and number of previous cesarean sections greater than parity (*n* = 243). Reasons are not mutually exclusive. ^a^Numbers provided by ISD Scotland. CHI, community health index; ISD, Information Services Division; NRS, National Records of Scotland; SMR02, Scottish Morbidity Record Maternity Inpatient and Day Case dataset; VBAC, vaginal birth after previous cesarean.

In total, 74,043 singleton term births to women with one or more previous cesarean sections were identified as meeting the study eligibility criteria (Fig 1). Of these, 28,464 (38.4%) were to women classified as having a planned VBAC, and 45,579 (61.6%) were to women classified as having an ERCS. The ERCS rate among this group of women increased each year during the study period, from 50.5% in 2002 to 72.4% in 2015. Table 1 shows the characteristics of the study population by planned mode of delivery. Women who had a planned VBAC were more likely than those who had an ERCS to be younger, born outside the UK, be sole registered mothers (no partner or husband registered on the birth certificate), and have a lower socioeconomic status. They were also more likely to have just one prior cesarean section, have had one or more prior vaginal deliveries, have a shorter interpregnancy interval, be smokers at booking for pregnancy care, have a hypertensive disorder, have prelabor rupture of membranes, have delivered at late term (39–41 weeks gestation), and have delivered an infant less than the 10th centile for birth weight. They were less likely than women who had an ERCS to be overweight or obese, to have diabetes, and to have delivered an infant more than the 90th centile for birth weight.

### Planned VBAC compared to ERCS

**Fig 2:**
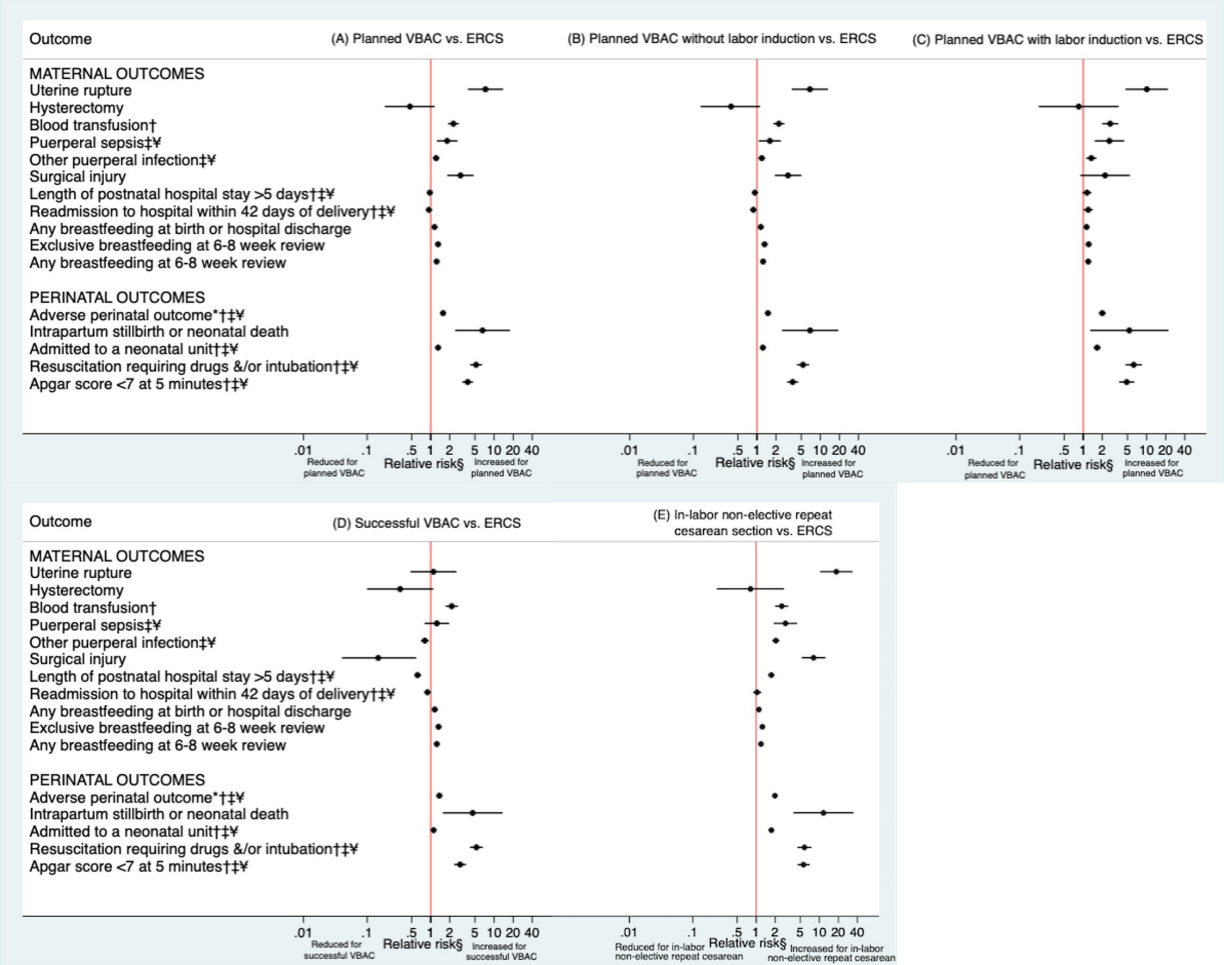
**Maternal and perinatal outcomes following (A) planned VBAC compared to ERCS, (B) planned VBAC without labor induction compared to ERCS, (C) planned VBAC with labor induction compared to ERCS, (D) successful VBAC compared to ERCS, (E) and in-labor nonelective repeat cesarean section compared to ERCS.** §Hysterectomy and intrapartum stillbirth or neonatal death only adjusted for year of delivery because of low number of events, whereas other outcomes were adjusted for year of delivery, sociodemographic factors (maternal age, mother’s country of birth, marital status/registration type, and socioeconomic status) and maternal medical and pregnancy-related factors (number of previous cesarean sections, any prior vaginal delivery, interpregnancy interval, maternal smoking status at booking, maternal BMI at booking, hypertensive disorder where † is shown, diabetes where ‡ is shown, and prelabor rupture of membranes where ¥ is shown). Breastfeeding and perinatal outcomes were additionally adjusted for infant-related factors (sex of infant, gestational age at delivery, and birth weight centile). *Includes intrapartum stillbirth or neonatal death, admission to a neonatal unit, resuscitation requiring drugs and/or intubation or an Apgar score < 7 at 5 minutes. ERCS, elective repeat cesarean section; VBAC, vaginal birth after previous cesarean.

Maternal and perinatal outcomes according to planned mode of delivery are shown in Table 2 and Fig 2A. Overall, 1.8% of those having a planned VBAC and 0.8% of those having an ERCS experienced serious maternal morbidity (uterine rupture, peripartum hysterectomy, blood transfusion, puerperal sepsis, or surgical injury), and 8.0% of the planned VBAC and 6.4% of the ERCS group experienced one or more of the adverse perinatal outcomes considered (intrapartum stillbirth or neonatal death, admission to a neonatal unit, resuscitation requiring drugs, and/or intubation or Apgar score < 7 at 5 minutes). Having only adjusted for year of delivery (base model), women who had a planned VBAC were significantly more likely than those delivering by ERCS to have uterine rupture, a blood transfusion, puerperal sepsis, and surgical injury. They were also significantly more likely to have an intrapartum stillbirth or neonatal death, a baby requiring resuscitation with drugs and/or intubating, a baby with an Apgar score of <7 at 5 minutes, and a baby experiencing a composite outcome comprising any of the adverse perinatal outcomes considered. However, in the base model, women who had a planned VBAC were significantly less likely than those delivering by ERCS to have a postnatal hospital stay greater than 5 days and were significantly less likely to be readmitted overnight to hospital within 42 days of birth. They were also more likely than those delivering by ERCS to breastfeed at birth or hospital discharge and at 6–8 weeks postpartum.

**Table 2:** Maternal and perinatal outcomes following planned VBAC compared to ERCS.

	ERCS *n* outcome events/total *n* (%)	Planned VBAC *n* outcome events/total *n* (%)	Base model^1^ relative risk (95% CI)	Model A^2^ relative risk (95% CI)	Model B^3^ relative risk (95% CI)	Model C^4^ relative risk (95% CI)
**Maternal outcomes**						
Uterine rupture	17/45,579 (0.04)	69/28,464 (0.24)	**6.26 (3.64–10.76)** ***p* < 0.001**	**6.28 (3.62–10.89)** ***p* < 0.001**	**7.33 (3.88–13.88)** ***p* < 0.001**	-
Peripartum hysterectomy	19/45,579 (0.04)	6/28,464 (0.02)	0.47 (0.19–1.16) *p* = 0.102	NC	NC	-
Blood transfusion†	226/45,579 (0.50)	324/28,464 (1.14)	**2.12 (1.78–2.52)** ***p* < 0.001**	**2.09 (1.76–2.49)** ***p* < 0.001**	**2.29 (1.88–2.79)** ***p* < 0.001**	-
Puerperal sepsis‡¥	78/45,579 (0.17)	76/28,464 (0.27)	**1.73 (1.25–2.38)** ***p* = 0.001**	**1.69 (1.22–2.33)** ***p* = 0.001**	**1.82 (1.25–2.65)** ***p* = 0.002**	-
Other puerperal infection‡¥	1,016/45,579 (2.23)	659/28,464 (2.32)	1.00 (0.90–1.11) *p* = 0.986	0.99 (0.89–1.10) *p* = 0.855	**1.22 (1.09–1.36)** ***p* < 0.001**	-
Surgical injury	40/45,579 (0.09)	# (0.17)	**1.96 (1.29–2.98)** ***p* = 0.002**	**2.01 (1.33–3.05)** ***p* = 0.001**	**2.96 (1.83–4.77)** ***p* < 0.001**	-
Length of postnatal hospital stay > 5 days†‡¥	1,410/45,579 (3.09)	877/28,464 (3.08)	**0.88 (0.81–0.97)** ***p* = 0.007**	**0.89 (0.82–0.98)** ***p* = 0.013**	0.97 (0.88–1.08) *p* = 0.574	-
Readmission to hospital within 42 days of birth^a^†‡¥	1,332/45,577 (2.92)	710/28,463 (2.49)	**0.88 (0.80–0.96)** ***p* = 0.006**	**0.87 (0.79–0.96)** ***p* = 0.004**	0.94 (0.84–1.04) *p* = 0.234	-
Any breastfeeding at birth or hospital discharge^b^	21,403/39,297 (54.46)	14,906/23,453 (63.56)	**1.18 (1.16–1.20)** ***p* < 0.001**	**1.19 (1.18–1.21)** ***p* < 0.001**	**1.18 (1.16–1.20)** ***p* < 0.001**	**1.15 (1.13–1.18)** ***p* < 0.001**
Exclusive breastfeeding at 6–8 week review^c^	9,788/39,251 (24.94)	8,085/24,090 (33.56)	**1.34 (1.31–1.38)** ***p* < 0.001**	**1.37 (1.34–1.41)** ***p* < 0.001**	**1.38 (1.34–1.41)** ***p* < 0.001**	**1.31 (1.27–1.35)** ***p* < 0.001**
Any breastfeeding at 6–8 week review^c^	13,556/39,251 (34.54)	10,496/24,090 (43.57)	**1.27 (1.24–1.30)** ***p* < 0.001**	**1.29 (1.26–1.31)** ***p* < 0.001**	**1.29 (1.26–1.32)** ***p* < 0.001**	**1.24 (1.21–1.27)** ***p* < 0.001**
**Perinatal outcomes**^**d**^						
Adverse perinatal outcome^e^†‡¥	2,570/40,369 (6.37)	2,049/25,658 (7.99)	**1.22 (1.15–1.30)** ***p* < 0.001**	**1.22 (1.15–1.30)** ***p* < 0.001**	**1.35 (1.26–1.44)** ***p* < 0.001**	**1.57 (1.46–1.68)** ***p* < 0.001**
Intrapartum stillbirth or neonatal death	5/45,567 (0.01)	20/28,449 (0.07)	**6.59 (2.43–17.87)** ***p* < 0.001**	NC	NC	NC
Admitted to a neonatal unit†‡¥	2,377/45,062 (5.27)	1,517/27,847 (5.45)	1.00 (0.93–1.07) *p* = 0.967	1.00 (0.94–1.07) *p* = 0.960	**1.11 (1.03–1.20)** ***p* = 0.006**	**1.31 (1.22–1.41)** ***p* < 0.001**
Resuscitation requiring drugs and&/or intubation†‡¥	133/40,830 (0.33)	429/26,295 (1.63)	**4.67 (3.84–5.68)** ***p* < 0.001**	**4.68 (3.84–5.70)** ***p* < 0.001**	**5.19 (4.17–6.45)** ***p* < 0.001**	**5.22 (4.19–6.50)** ***p* < 0.001**
Apgar score < 7 at 5 minutes†‡¥	192/45,194 (0.42)	405/28,092 (1.44)	**3.45 (2.90–4.09)** ***p* < 0.001**	**3.46 (2.91–4.11)** ***p* < 0.001**	**3.66 (3.01–4.46)** ***p* < 0.001**	**3.84 (3.15–4.68)** ***p* < 0.001**

Bold text indicates statistically significant findings at the 5% level.

1 Base model adjusted for year of delivery.

2 Model A adjusted for year of delivery and sociodemographic factors (maternal age, mother’s country of birth, marital status/registration type, and socioeconomic status).

3 Model B adjusted for variables in model A and additionally adjusted for maternal medical and pregnancy-related factors (number of previous cesarean sections, any prior vaginal delivery, interpregnancy interval, maternal smoking status at booking, maternal BMI at booking, hypertensive disorder where † is shown, diabetes where ‡ is shown, and prelabor rupture of membranes where ¥ is shown).

4 Model C adjusted for variables in model B and additionally adjusted for infant-related factors (sex of infant, gestational age at delivery, and birth weight centile).

#Numbers have not been shown to protect against potential disclosure risks.

^a^Women who died before discharge or were not discharged within 42 days of birth excluded from analysis of overnight readmission to hospital (*n* = 3).

^b^Intrapartum stillbirths (*n* = 6) and births missing data on feeding at birth and hospital discharge (*n* = 11,287, 15.2%) were excluded from analysis of breastfeeding at birth or hospital discharge.

^c^Intrapartum stillbirths (*n* = 6), neonatal deaths (46), and births missing infant feeding data at 6–8 week review (*n* = 10,650, 14.4%) were excluded from analysis of breastfeeding outcomes at 6–8 weeks.

^d^All perinatal outcomes exclude deaths due to congenital abnormalities (*n* = 27) and any remaining intrapartum stillbirths (*n* = 5) and births missing the outcome in question (*n* = 1,102, 1.5% for admission to a neonatal unit; *n* = 6,886, 9.3% for resuscitation; *n* = 725, 1.0% for Apgar score) additionally excluded from analysis of neonatal unit admission, resuscitation, and Apgar score.

^e^Includes intrapartum stillbirth or neonatal death, admission to a neonatal unit, resuscitation requiring drugs and/or intubation, or an Apgar score < 7 at 5 minutes.

Abbreviations: ERCS, elective repeat cesarean section; NC, not calculated because of low number of events; VBAC, vaginal birth after previous cesarean

Adjustment for sociodemographic factors (model A) essentially did not change the findings, whereas further adjustment for maternal medical and pregnancy-related factors (model B) made a difference to some of the effect estimates. In particular, the effect of planned mode of delivery on length of postnatal hospital stay and overnight readmission to hospital was attenuated and not statistically significant after adjusting for maternal medical and pregnancy-related factors. However, a significant interaction between planned mode of delivery and any prior vaginal delivery was found for these outcomes in the fully adjusted models (*p* < 0.001 and *p* = 0.002 for length of postnatal hospital stay and overnight readmission to hospital, respectively): planned VBAC compared to ERCS was associated with significantly reduced odds of having either a postnatal hospital stay greater than 5 days or an overnight readmission to hospital within 42 days of birth in women with any prior vaginal deliveries (adjusted OR [aOR] 0.70, 95% CI 0.59–0.84, *p* < 0.001 and aOR 0.74, 95% CI 0.61–0.89, *p* = 0.001, respectively), but there was no significant association in women with no prior vaginal deliveries (aOR 1.10, 95% CI 0.98–1.23, *p* = 0.093 and aOR 1.03, 95% CI 0.92–1.16, *p* = 0.618 respectively). The corresponding absolute risks of having a postnatal hospital stay greater than 5 days for planned VBAC compared to ERCS was 2.74% versus 3.67% in women with any prior vaginal deliveries and 3.30% versus 2.97% in women without any prior vaginal deliveries. The absolute risks of having an overnight readmission to hospital within 42 days of birth for planned VBAC compared to ERCS was 2.19% versus 3.21% in women with any prior vaginal deliveries and 2.67% versus 2.87% in women without any prior vaginal deliveries. There was also evidence of an interaction (*p* = 0.003) between planned mode of delivery and number of prior cesarean sections for the outcome postnatal hospital stay greater than 5 days in the fully adjusted model: planned VBAC compared to ERCS was associated with significantly increased odds of this outcome in women with two or more prior cesarean sections (aOR 1.47, 95% CI 1.10–1.98, *p* = 0.010) but no significant difference in women with one prior cesarean section (aOR 0.92, 95% CI 0.83–1.03, *p* = 0.143), with the corresponding absolute risks of this outcome for planned VBAC compared to ERCS 5.76% versus 3.91% in women with two or more prior cesarean sections and 2.99% versus 2.82% in women with one prior cesarean section.

Adjustment for maternal medical and pregnancy-related factors made little difference to the effect estimates for the other outcomes considered with the exception of those for other puerperal infection and admission to a neonatal unit, which were significantly increased in the planned VBAC group following this adjustment. However, for the outcome other puerperal infection, a significant interaction (*p* < 0.001) was detected between planned mode of delivery and any prior vaginal delivery in the fully adjusted model: planned VBAC compared to ERCS was associated with significantly increased odds of other puerperal infection in those without any prior vaginal deliveries (aOR 1.38, 95% CI 1.22–1.55, *p* < 0.001) but significantly reduced odds of other puerperal infection in those with any prior vaginal deliveries (aOR 0.74, 95% CI 0.59–0.93, *p* = 0.011), with the corresponding absolute risks of this outcome for planned VBAC compared to ERCS 2.89% versus 2.28% in women without any prior vaginal deliveries and 1.41% versus 1.97% in women with any prior vaginal deliveries. The effect of planned mode of delivery on breastfeeding outcomes and perinatal outcomes remained after further adjustment for infant-related factors (model C). In the fully adjusted model, there was evidence (*p* < 0.001) that the effect of planned VBAC on breastfeeding at birth or hospital discharge was stronger in women without prior vaginal deliveries (adjusted RR [aRR] 1.17, 95% CI 1.15–1.19, *p* < 0.001) than in women with any prior vaginal deliveries (aRR 1.09, 95% CI 1.06–1.12, *p* < 0.001). No other significant interactions were found. The absolute risk of third- or fourth-degree perineal tears in the planned VBAC group was 2.9%.

### Planned VBAC with and without labor induction compared to ERCS

Of the women who had a planned VBAC, 18.5% (5,245/28,364) had their labor induced. A combination of surgical and medical induction was the main method used to induce labor (42.9% of inductions) with surgical or medical methods alone used less frequently (27.5% and 28.7% of inductions, respectively). Maternal and perinatal outcomes according to whether planned VBAC was attempted with and without labor induction compared to ERCS are shown in Table 3 and Fig 2C and 2B, respectively. Essentially, the findings largely mirror those reported for planned VBAC compared to ERCS. Women who had a planned VBAC with and those who had a planned VBAC without labor induction were both more likely than women delivering by ERCS to have uterine rupture, a blood transfusion, puerperal sepsis, or other puerperal infection and experience all adverse perinatal outcomes considered, at least in the fully adjusted models. The effect estimates for these outcomes were consistently highest in the women who had planned VBAC with labor induction. Women who had a planned VBAC with labor induction were also more likely than those delivering by ERCS to be readmitted overnight to hospital within 42 days of birth. In contrast, women who had a planned VBAC without labor induction were less likely than those delivering by ERCS to be readmitted. However, the odds of having surgical injury were only significantly raised in women who had planned VBAC without labor induction, noting the greater power of this analysis. Both groups of women were more likely to breastfeed at birth or hospital discharge and at 6–8 week postpartum than those delivering by ERCS.

**Table 3:** Maternal and perinatal outcomes following planned VBAC with and without labor induction compared to ERCS.

	ERCS	Planned VBAC without labor induction					Planned VBAC with labor induction				
	*n* outcome events/total *N* (%)	*n* outcome events/total *N* (%)	Base model^1^ relative risk (95% CI)	Model A^2^ relative risk (95% CI)	Model B^3^ relative risk (95% CI)	Model C^4^ relative risk (95% CI)	*n* outcome events/ total *N* (%)	Base model^1^ relative risk (95% CI)	Model A^2^ relative risk (95% CI)	Model B^3^ relative risk (95% CI)	Model C^4^ relative risk (95% CI)
**Maternal outcomes**											
Uterine rupture	17/45,579 (0.04)	53/23,119 (0.23)	**5.92** **(3.38–10.37)** ***p* < 0.001**	**5.89** **(3.34–10.41)** ***p* < 0.001**	**6.85** **(3.55–13.19)** ***p* < 0.001**	-	16/5,245 (0.31)	**7.89** **(3.99–15.61)** ***p* < 0.001**	**8.17** **(4.08–16.35)** ***p* < 0.001**	**10.06** **(4.65–21.72)** ***p* < 0.001**	-
Peripartum hysterectomy	19/45,579 (0.04)	# (0.02)	0.39 (0.13–1.12) *p* = 0.080	NC	NC	-	#	0.85 (0.20–3.64) *p* = 0.828	NC	NC	-
Blood transfusion†	226/45,579 (0.50)	256/23,119 (1.11)	**2.06** **(1.72–2.47)** ***p* < 0.001**	**2.04** **(1.70–2.45)** ***p* < 0.001**	**2.22** **(1.81–2.72)** ***p* < 0.001**	-	68/5,245 (1.30)	**2.42** **(1.83–3.19)** ***p* < 0.001**	**2.38** **(1.81–3.14)** ***p* < 0.001**	**2.67** **(1.99–3.58)** ***p* < 0.001**	-
Puerperal sepsis‡¥	78/45,579 (0.17)	54/23,119 (0.23)	**1.51** **(1.06–2.14)** ***p* = 0.021**	**1.48** **(1.04–2.10)** ***p* = 0.028**	**1.60** **(1.07–2.39)** ***p* = 0.021**	-	21/5,245 (0.40)	**2.55** **(1.56–4.16)** ***p* < 0.001**	**2.49** **(1.53–4.06)** ***p* < 0.001**	**2.59** **(1.52–4.44)** ***p* < 0.001**	-
Other puerperal infection‡¥	1,016/45,579 (2.23)	524/23,119 (2.27)	0.98 (0.88–1.10) *p* = 0.757	0.97 (0.87–1.09) *p* = 0.639	**1.19** **(1.06–1.34)** ***p* = 0.035**	-	135/5,245 (2.57)	1.10 (0.91–1.32) *p* = 0.325	1.08 (0.90–1.30) *p* = 0.415	**1.34** **(1.11–1.62)** ***p* = 0.027**	-
Surgical injury	40/45,579 (0.09)	42/23,119 (0.18)	**2.12** **(1.38–3.25)** ***p* < 0.001**	**2.17** **(1.42–3.32)** ***p* < 0.001**	**3.10** **(1.91–5.04)** ***p* < 0.001**	-	#	1.33 (0.56–3.15) *p* = 0.518	1.37 (0.58–3.26) *p* = 0.470	2.22 (0.90–5.47) *p* = 0.082	-
Length of postnatal hospital stay > 5 days†‡¥	1,410/45,579 (3.09)	672/23,119 (2.91)	**0.83** **(0.76–0.92)** ***p* < 0.001**	**0.84** **(0.77–0.93)** ***p* = 0.001**	0.93 (0.83–1.04) *p* = 0.120	-	202/5,245 (3.85)	1.11 (0.96–1.30) *p* = 0.167	1.11 (0.95–1.29) *p* = 0.189	1.15 (0.97–1.35) *p* = 0.099	-
Readmission to hospital within 42 days of birth^a^†‡¥	1,332/45,577 (2.92)	531/23,118 (2.30)	**0.81** **(0.73–0.89)** ***p* < 0.001**	**0.80** **(0.72–0.89)** ***p* < 0.001**	**0.88** **(0.78–0.98)** ***p* = 0.024**	-	176/5,245 (3.36)	**1.19** **(1.01–1.39)** ***p* = 0.036**	**1.18** **(1.00–1.38)** ***p* = 0.045**	**1.20** **(1.01–1.42)** ***p* = 0.035**	-
Any breastfeeding at birth or hospital discharge^b^	21,403/39,297 (54.46)	12,187/19,083 (63.86)	**1.19** **(1.17–1.20)** ***p* < 0.001**	**1.19** **(1.17–1.21)** ***p* < 0.001**	**1.18** **(1.16–1.20)** ***p* < 0.001**	**1.15** **(1.13–1.17)** ***p* < 0.001**	2,664/4,292 (62.07)	**1.15** **(1.12–1.18)** ***p* < 0.001**	**1.20** **(1.17–1.23)** ***p* < 0.001**	**1.17** **(1.14–1.21)** ***p* < 0.001**	**1.13** **(1.09–1.16)** ***p* < 0.001**
Exclusive breastfeeding at 6–8 week review^c^	9,788/39,251 (24.94)	6,720/19,531 (34.41)	**1.38** **(1.34–1.42)** ***p* < 0.001**	**1.39** **(1.36–1.43)** ***p* < 0.001**	**1.38** **(1.34–1.42)** ***p* < 0.001**	**1.32** **(1.28–1.36)** ***p* < 0.001**	1,339/4,471 (29.9)	**1.20** **(1.14–1.26)** ***p* < 0.001**	**1.29** **(1.24–1.35)** ***p* < 0.001**	**1.33** **(1.26–1.41)** ***p* < 0.001**	**1.22** **(1.16–1.30)** ***p* < 0.001**
Any breastfeeding at 6–8 week review^c^	13,556/39,251 (34.54)	8,663/19,531 (44.36)	**1.29** **(1.26–1.32)** ***p* < 0.001**	**1.29** **(1.27–1.32)** ***p* < 0.001**	**1.29** **(1.26–1.32)** ***p* < 0.001**	**1.25** **(1.22–1.28)** ***p* < 0.001**	1,788/4,471 (40.24)	**1.17** **(1.13–1.22)** ***p* < 0.001**	**1.25** **(1.21–1.30)** ***p* < 0.001**	**1.28** **(1.22–1.34)** ***p* < 0.001**	**1.20** **(1.14–1.25)** ***p* < 0.001**
**Perinatal outcomes**^**d**^											
Adverse perinatal outcome^e^†‡¥	2,570/40,369 (6.37)	1,583/20,878 (7.58)	**1.15** **(1.08–1.23)** ***p* < 0.001**	**1.16** **(1.08–1.24)** ***p* < 0.001**	**1.30** **(1.20–1.39)** ***p* < 0.001**	**1.49** **(1.38–1.60)** ***p* < 0.001**	458/4,724 (9.70)	**1.51** **(1.36–1.68)** ***p* < 0.001**	**1.49** **(1.34–1.66)** ***p* < 0.001**	**1.56** **(1.40–1.75)** **P<0.001**	**2.00** **(1.78–2.25)** ***p* < 0.001**
Intrapartum stillbirth or neonatal death	5/45,567 (0.01)	(0.07)	**6.9** **(2.48–19.19)** ***p* < 0.001**	NC	NC	NC	# (0.06)	**5.35** **(1.29–22.12)** ***p* = 0.021**	NC	NC	NC
Admitted to a neonatal unit†‡¥	2,377/45,062 (5.27)	1,171/22,645 (5.17)	0.94 (0.88–1.02) *p* = 0.126	0.95 (0.88–1.02) *p* = 0.182	1.07 (0.99–1.16) *p* = 0.083	**1.24** **(1.15–1.35)** ***p* < 0.001**	340/5,136 (6.62)	**1.23** **(1.09–1.39)** ***p* < 0.001**	**1.21** **(1.08–1.37)** ***p* = 0.002**	**1.27** **(1.12–1.45)** ***p* < 0.001**	**1.66** **(1.46–1.89)** ***p* < 0.001**
Resuscitation requiring drugs and/or intubation†‡¥	133/40,830 (0.33)	335/21,405 (1.57)	**4.69** **(3.83–5.75)** ***p* < 0.001**	**4.72** **(3.85–5.78)** ***p* < 0.001**	**5.30** **(4.24–6.62)** ***p* < 0.001**	**5.34** **(4.27–6.68)** ***p* < 0.001**	91/4,824 (1.89)	**5.66** **(4.32–7.40)** ***p* < 0.001**	**5.59** **(4.27–7.33)** ***p* < 0.001**	**6.17** **(4.60–8.27)** ***p* < 0.001**	**6.27** **(4.66–8.45)** ***p* < 0.001**
Apgar score < 7 at 5 minutes†‡¥	192/45,194 (0.42)	309/22,807 (1.35)	**3.24** **(2.70–3.88)** ***p* < 0.001**	**3.26** **(2.72–3.91)** ***p* < 0.001**	**3.50** **(2.85–4.29)** ***p* < 0.001**	**3.66** **(2.99–4.50)** ***p* < 0.001**	95/5,190 (1.83)	**4.39** **(3.43–6.62)** ***p* < 0.001**	**4.31** **(3.37–5.53)** ***p* < 0.001**	**4.47** **(3.41–5.86)** ***p* < 0.001**	**4.87** **(3.69–6.43)** ***p* < 0.001**

Bold text indicates statistically significant findings at the 5% level.

1 Base model adjusted for year of delivery.

2 Model A adjusted for year of delivery and sociodemographic factors (maternal age, mother’s country of birth, marital status/registration type, and socioeconomic status).

3 Model B adjusted for variables in model A and additionally adjusted for maternal medical and pregnancy-related factors (number of previous cesarean sections, any prior vaginal delivery, interpregnancy interval, maternal smoking status at booking, maternal BMI at booking, hypertensive disorder where † is shown, diabetes where ‡ is shown, and prelabor rupture of membranes where ¥ is shown).

4 Model C adjusted for variables in model B and additionally adjusted for infant-related factors (sex of infant, gestational age at delivery, and birth weight centile).

^a^Women who died before discharge or were not discharged within 42 days of birth were excluded from analysis of overnight readmission to hospital (*n* = 3).

^b^Intrapartum stillbirths (*n* = 6) and births missing data on feeding at birth and hospital discharge (*n* = 11,265, 15.2%) were excluded from analysis of breastfeeding at birth or hospital discharge.

^c^Intrapartum stillbirths (*n* = 6), neonatal deaths (46), and births missing infant feeding data at 6–8 week review (*n* = 10,638, 14.4%) were excluded from analysis of breastfeeding outcomes at 6–8 weeks.

^d^All perinatal outcomes exclude deaths because of congenital abnormalities (*n* = 27) and any remaining intrapartum stillbirths (*n* = 5) and births missing the outcome in question (*n* = 1,068, 1.4% for admission to a neonatal unit; *n* = 6,852, 9.3% for resuscitation; *n* = 720, 1.0% for Apgar score) were additionally excluded from analysis of neonatal unit admission, resuscitation, and Apgar score.

^e^Includes intrapartum stillbirth or neonatal death, admission to a neonatal unit, resuscitation requiring drugs, and/or intubation or an Apgar score < 7 at 5 minutes.

#Numbers or numbers and percentages have not been shown to protect against potential disclosure risks.

Abbreviations: CI, confidence interval; ERCS, elective repeat cesarean section; NC, not calculated because of low number of events; VBAC, vaginal birth after previous cesarean

### Successful VBAC and in-labor nonelective repeat cesarean section compared to ERCS

Of the women who had a planned VBAC, 71.6% (20,375/28,464) had a successful VBAC. Maternal and perinatal outcomes following either successful VBAC or in-labor nonelective repeat cesarean section compared with ERCS are shown in Table 4 and Fig 2D and 2E, respectively. Women delivering by in-labor nonelective repeat cesarean section were significantly more likely than those delivering by ERCS to experience all adverse outcomes considered, with the exception of peripartum hysterectomy and overnight readmission to hospital within 42 days of birth. Women who had a successful VBAC were more likely than those delivering by ERCS to have a blood transfusion and experience all adverse perinatal outcomes considered, at least in the fully adjusted models. However, they were less likely than those delivering by ERCS to have other puerperal infection, surgical injury, or a postnatal hospital stay of greater than 5 days. Breastfeeding at birth or hospital discharge and at 6–8 weeks postpartum were both more likely in women delivering by successful VBAC and those delivering by in-labor nonelective repeat cesarean section compared to those delivering by ERCS.

**Table 4:** Maternal and perinatal outcomes following successful VBAC and in-labor nonelective repeat cesarean section compared to ERCS.

	ERCS	Successful VBAC					In-labor nonelective repeat cesarean section				
	*n* outcome events/total *N* (%)	*n* outcome events/total *N* (%)	Base model^1^ relative risk (95% CI)	Model A^2^ relative risk (95% CI)	Model B^3^ relative risk (95% CI)	Model C^4^ relative risk (95% CI)	*n* outcome events/total *N* (%)	Base model^1^ relative risk (95% CI)	Model A^2^ relative risk (95% CI)	Model B^3^ relative risk (95% CI)	Model C^4^ relative risk (95% CI)
**Maternal outcomes**											
Uterine rupture	17/45,579 (0.04)	9/20,375 (0.04)	1.14 (0.51–2.56) *p* = 0.744	1.16 (0.51–2.60) *p* = 0.727	1.10 (0.48–2.56) *p* = 0.820	-	60/8,089 (0.74)	**19.28** **(11.09–33.53)** ***p* < 0.001**	**19.28** **(10.97–33.88)** ***p* < 0.001**	**18.58** **(10.30–33.53)** ***p* < 0.001**	-
Peripartum hysterectomy	19/45,579 (0.04)	# (0.01)	0.33 (0.10–1.10) *p* = 0.071	NC	NC	-	#	0.82 (0.24–2.76) *p* = 0.752	NC	NC	-
Blood transfusion†	226/45,579 (0.50)	213/20,375 (1.05)	**1.95** **(1.61–2.36)** ***p* <0.001**	**1.93** **(1.59–2.34)** ***p* < 0.001**	**2.15** **(1.72–2.69)** ***p* < 0.001**	-	111/8,089 (1.37)	**2.54** **(2.02–3.21**) ***p* < 0.001**	**2.51** **(1.99–3.17**) ***p* < 0.001**	**2.55** **(2.00–3.24)** ***p* < 0.001**	-
Puerperal sepsis‡¥	78/45,579 (0.17)	37/20,375 (0.18)	1.17 (0.79–1.73) *p* = 0.430	1.15 (0.78–1.70) *p* = 0.489	1.25 (0.80–1.96) *p* = 0.319	-	39/8,089 (0.48)	**3.14** **(2.12–4.66)** ***p* < 0.001**	**3.08** **(2.08–4.57)** ***p* < 0.001**	**2.92** **(1.91–4.46)** ***p* < 0.001**	-
Other puerperal infection‡¥	1,016/45,579 (2.23)	312/20,375 (1.53)	**0.66** **(0.58–0.75**) ***p* < 0.001**	**0.65** **(0.57–0.74**) ***p* < 0.001**	**0.81** **(0.70–0.93)** ***p* = 0.004**	-	347/8,089 (4.29)	**1.88** **(1.65–2.13**) ***p* < 0.001**	**1.87** **(1.65–2.13**) ***p* < 0.001**	**2.06** **(1.80–2.35)** ***p* < 0.001**	-
Surgical injury	40/45,579 (0.09)	# (0.01)	**0.11** **(0.03–0.47**) ***p* = 0.003**	**0.12** **(0.03–0.48**) ***p* = 0.003**	**0.15** **(0.04–0.59)** ***p* = 0.007**	-	46/8,089 (0.57)	**6.67** **(4.37–10.18)** ***p* < 0.001**	**6.92** **(4.56–10.49)** ***p* < 0.001**	**8.12** **(5.26–12.56)** ***p* < 0.001**	-
Length of postnatal hospital stay > 5 days†‡¥	1,410/45,579 (3.09)	427/20,375 (2.10)	**0.60** **(0.53–0.67**) ***p* < 0.001**	**0.61** **(0.53–0.67**) ***p* < 0.001**	**0.62** **(0.55–0.71)** ***p* < 0.001**	-	450/8,089 (5.56)	**1.63** **(1.46–1.82**) ***p* < 0.001**	**1.67** **(1.50–1.87**) ***p* < 0.001**	**1.74** **(1.55–1.96)** ***p* < 0.001**	-
Readmission to hospital within 42 days of birth^a^†‡¥	1,332/45,577 (2.92)	478/20,374 (2.35)	**0.82** **(0.74–0.92**) ***p* < 0.001**	**0.82** **(0.73–0.91**) ***p* < 0.001**	0.89 (0.79–1.01) *p* = 0.061	-	232/8,089 (2.87)	1.02 (0.88–1.17) *p* = 0.835	1.01 (0.88–1.16) *p* = 0.899	1.04 (0.90–1.21) *p* = 0.611	-
Any breastfeeding at birth or hospital discharge^b^	21,403/39,297 (54.46)	10,444/16,616 (62.86)	**1.17** **(1.15–1.19**) ***p* < 0.001**	**1.19** **(1.17–1.21**) ***p* < 0.001**	**1.19** **(1.17–1.21)** ***p* < 0.001**	**1.16** **(1.14–1.18)** ***p* < 0.001**	4,462/6,837 (65.26)	**1.21** **(1.19–1.24**) ***p* < 0.001**	**1.20** **(1.18–1.22**) ***p* < 0.001**	**1.14** **(1.11–1.17)** ***p* < 0.001**	**1.11** **(1.08–1.14)** ***p* < 0.001**
Exclusive breastfeeding at 6–8 week review^c^	9,788/39,251 (24.94)	5,723/17,046 (33.57)	**1.34** **(1.30–1.38**) ***p* < 0.001**	**1.40** **(1.36–1.44**) ***p* < 0.001**	**1.40** **(1.35–1.44)** ***p* < 0.001**	**1.33** **(1.29–1.37)** ***p* < 0.001**	2,362/7,044 (33.53)	**1.34** **(1.29–1.39**) ***p* < 0.001**	**1.32** **(1.27–1.37**) ***p* < 0.001**	**1.32** **(1.27–1.38)** ***p* < 0.001**	**1.26** **(1.21–1.31)** ***p* < 0.001**
Any breastfeeding at 6–8 week review^c^	13,556/39,251 (34.54)	7,336/17,046 (43.04)	**1.25** **(1.22–1.28**) ***p* < 0.001**	**1.29** **(1.26–1.32**) ***p* < 0.001**	**1.30** **(1.27–1.34)** ***p* < 0.001**	**1.25** **(1.22–1.29)** ***p* < 0.001**	3,160/7,044 (44.86)	**1.31** **(1.27–1.35**) ***p* < 0.001**	**1.27** **(1.24–1.31**) ***p* < 0.001**	**1.25** **(1.21–1.30)** ***p* < 0.001**	**1.20** **(1.16–1.24)** ***p* < 0.001**
**Perinatal outcomes**^**d**^											
Adverse perinatal outcome^e^†‡¥	2,570/40,369 (6.37)	1,298/18,385 (7.06)	1.07 (1.00–1.15) *p* = 0.065	1.06 (0.99–1.14) *p* = 0.085	**1.17** **(1.08–1.27)** ***p* < 0.001**	**1.37** **(1.26–1.48)** ***p* < 0.001**	751/7,273 (10.33)	**1.61** **(1.48–1.76**) ***p* < 0.001**	**1.65** **(1.51–1.80**) ***p* < 0.001**	**1.72** **(1.57–1.89)** ***p* < 0.001**	**1.99** **(1.82–2.19)** ***p* < 0.001**
Intrapartum stillbirth or neonatal death	5/45,567 (0.01)	10/20,365 (0.05)	**4.60** **(1.55–13.64**) ***p* = 0.006**	NC	NC	NC	10/8,084 (0.12)	**11.63** **(3.89–34.75**) ***p* < 0.001**	NC	NC	NC
Admitted to a neonatal unit†‡¥	2,377/45,062 (5.27)	937/20,014 (4.68)	**0.85** **(0.79–0.92)** ***p* < 0.001**	**0.85** **(0.78–0.92)** ***p* < 0.001**	0.94 (0.86–1.03) *p* = 0.182	**1.11** **(1.01–1.21)** ***p* = 0.023**	580/7,833 (7.40)	**1.38** **(1.26–1.52**) ***p* < 0.001**	**1.42** **(1.29–1.56**) ***p* < 0.001**	**1.49** **(1.35–1.65)** ***p* < 0.001**	**1.75** **(1.58–1.93)** ***p* < 0.001**
Resuscitation requiring drugs and/or intubation†‡¥	133/40,830 (0.33)	290/18,790 (1.54)	**4.62** **(3.76–5.68)** ***p* < 0.001**	**4.61** **(3.74–5.67)** ***p* < 0.001**	**5.24** **(4.15–6.61)** ***p* < 0.001**	**5.28** **(4.18–6.67)** ***p* < 0.001**	139/7,505 (1.85)	**5.58** **(4.39–7.09)** ***p* < 0.001**	**5.70** **(4.48–7.26)** ***p* < 0.001**	**5.85** **(4.54–7.53)** ***p* < 0.001**	**5.83** **(4.51–7.52)** ***p* < 0.001**
Apgar score < 7 at 5 minutes†‡¥	192/45,194 (0.42)	232/20,094 (1.15)	**2.76** **(2.28–3.33**) ***p* < 0.001**	**2.74** **(2.26–3.32**) ***p* < 0.001**	**2.78** **(2.25–3.45)** ***p* < 0.001**	**2.92** **(2.35–3.62)** ***p* < 0.001**	173/7,998 (2.16)	**5.22** **(4.24–6.42**) ***p* < 0.001**	**5.36** **(4.35–6.60**) ***p* < 0.001**	**5.39** **(4.33–6.72)** ***p* < 0.001**	**5.64** **(4.52–7.04)** ***p* < 0.001**

Bold text indicates statistically significant findings at the 5% level.

1 Base model adjusted for year of delivery.

2 Model A adjusted for year of delivery and sociodemographic factors (maternal age, mother’s country of birth, marital status/registration type, and socioeconomic status).

3 Model B adjusted for variables in model A and additionally adjusted for maternal medical and pregnancy-related factors (number of previous cesarean sections, any prior vaginal delivery, interpregnancy interval, maternal smoking status at booking, maternal BMI at booking, hypertensive disorder where † is shown, diabetes where ‡ is shown, and prelabor rupture of membranes where ¥ is shown).

4 Model C adjusted for variables in model B and additionally adjusted for infant-related factors (sex of infant, gestational age at delivery, and birth weight centile).

^a^Women who died before discharge or were not discharged within 42 days of birth were excluded from analysis of overnight readmission to hospital (*n* = 3).

^b^Intrapartum stillbirths (*n* = 6) and births missing data on feeding at birth and hospital discharge (*n* = 11,287, 15.2%) were excluded from analysis of breastfeeding at birth or hospital discharge.

^c^Intrapartum stillbirths (*n* = 6), neonatal deaths (46), and births missing infant feeding data at 6–8 week review (*n* = 10,650, 14.4%) were excluded from analysis of breastfeeding outcomes at 6–8 weeks.

^d^All perinatal outcomes exclude deaths due to congenital abnormalities (*n* = 27) and any remaining intrapartum stillbirths (*n* = 5) and births missing the outcome in question (*n* = 1,102, 1.5% for admission to a neonatal unit; *n* = 6,886, 9.3% for resuscitation; *n* = 725, 1.0% for Apgar score) were additionally excluded from analysis of neonatal unit admission, resuscitation, and Apgar score.

^e^Includes intrapartum stillbirth or neonatal death, admission to a neonatal unit, resuscitation requiring drugs and/or intubation, or an Apgar score < 7 at 5 minutes.

#Numbers or numbers and percentages have not been shown to protect against potential disclosure risks.

Abbreviations: CI, confidence interval; ERCS, elective repeat cesarean section; NC, not calculated because of low number of events; VBAC, vaginal birth after previous cesarean

### Sensitivity analyses

Similar effect estimates were obtained from the complete case analyses (S2 Table, S3 Table, and S4 Table) compared to those obtained using imputed data for the partially observed covariates, although the effect of planned VBAC on puerperal sepsis and other puerperal infection were not statistically significant in the fully adjusted complete case analysis, noting the more limited power of this analysis. In addition, the effect of planned VBAC without labor induction on maternal overnight readmission to hospital and the effect of in-labor nonelective repeat cesarean section on puerperal sepsis was not statistically significant in the fully adjusted complete case analysis, again noting the more limited power of this analysis. Confining the analysis to births delivered at 39 or more weeks’ gestation resulted in little change in the effect estimates, although the effect of planned VBAC without labor induction on maternal overnight readmission to hospital and the effect of VBAC on other puerperal infection was not statistically significant in the fully adjusted models. By contrast, the effect of planned VBAC with labor induction on the mother’s odds of having a postnatal stay greater than 5 days was only statistically significantly increased when the analysis was confined to births delivered at 39 or more weeks’ gestation (S5 Table, S6 Table, and S7 Table). Identifying intrapartum stillbirths and neonatal deaths, excluding deaths from congenital abnormalities from SSBID rather than NRS deaths, resulted in very similar effect estimates (S8 Table).

## Discussion

### Main findings

This population-based cohort study of term singleton births in Scotland suggests that among women considered eligible to have a planned VBAC, planned VBAC compared to ERCS is associated with an increased risk of the mother having uterine rupture, a blood transfusion, puerperal sepsis, and surgical injury as well as an increased risk of adverse perinatal outcomes. On the other hand, planned VBAC is associated with an increased likelihood of breastfeeding at birth or hospital discharge and at 6–8 weeks postpartum, whereas the association with the mother’s risk of having a postnatal hospital stay greater than 5 days, an overnight readmission to hospital within 42 days of birth, and other puerperal infection appears to differ according to whether she has any prior vaginal deliveries. In particular, the risk of these outcomes is reduced among women having a planned VBAC if they have any prior vaginal deliveries and is either not significantly different or increased in the case of other puerperal infection among those having a planned VBAC without any prior vaginal deliveries. There is also evidence that planned VBAC is associated with an increased risk of the mother having a postnatal hospital stay greater than 5 days among women with two or more prior cesarean sections but not among women with just one prior cesarean. Although there are significant differences in short-term outcomes between women who have a planned VBAC and those who have an ERCS, the absolute risk of adverse maternal and perinatal outcomes is small for either delivery approach. Overall, just 1.8% of those having a planned VBAC and 0.8% of those having an ERCS experienced serious maternal morbidity (uterine rupture, peripartum hysterectomy, blood transfusion, puerperal sepsis, or surgical injury), and 8.0% of the planned VBAC and 6.4% of the ERCS group experienced one or more of the adverse perinatal outcomes considered. Most maternal morbidity in the planned VBAC group occurred among those who needed an in-labor nonelective repeat cesarean section.

### Comparison with other studies

The risk of uterine rupture associated with planned VBAC is well known, although reported risk estimates vary widely between studies, which is likely to reflect differences in study methodology, case definitions used, or differences in the study populations such as the proportion of women who underwent induction of labor [16,34]. Although our absolute risk estimates for uterine rupture are lower than frequently quoted rates [16], they are comparable to several population-based studies [28,35,36], including a UK national study we performed that used validated case criteria [28]. Consistent with the findings of a systematic review conducted by the US Agency for Healthcare Research and Quality (AHRQ) of the literature between 1980 and September 2009 [16], and comparable to a number of subsequent predominately small studies [37–42], we found no significant difference in the risk of hysterectomy between women who had a planned VBAC and those who had an ERCS. However, we did find evidence that planned VBAC is associated with an increased risk of blood transfusion. The AHRQ review [16] also found evidence that planned VBAC is associated with an increased risk of blood transfusion, but only when the meta-analysis was confined to the four identified studies of women delivered at term rather than any gestational age (pooled estimates: 0.7% for planned VBAC versus 0.5% for ERCS, RR 1.30, 95% CI 1.15–1.47). Since the AHRQ review, although a number of studies have found no significant difference in the risk of blood transfusion between women who had planned VBAC and those that had an ERCS [36,40,42,43], a few of the larger studies have reported an elevated risk of this outcome for planned VBAC [39,44,45].

The AHRQ review reported that the overall risk of any type of maternal infection was not significantly different following planned VBAC compared to ERCS [16]. However, significant heterogeneity was apparent among the mostly small single-center studies identified, and the AHRQ review regarded the strength of evidence on maternal infection overall to be low because of the variability in how infection was defined and because the evidence was considered to be indirect and to have a high risk of bias. Although a more recent population-based study conducted in the UK reported that the risk of severe maternal sepsis did not significantly differ with planned VBAC compared to ERCS [41], a large population-based study conducted in the US found an elevated risk of puerperal sepsis and major puerperal infection with planned VBAC [45], consistent with our overall findings. However, neither of these prior studies examined whether any associations were modified by prior vaginal delivery. Our study suggests that prior vaginal delivery modifies the relationship between planned mode of delivery and other puerperal infection such that planned VBAC compared to ERCS is associated with an increased risk of this outcome in women without any prior vaginal delivery but with a reduced risk of this outcome in those with any prior vaginal deliveries. We also found evidence that the mother’s risk of having either a postnatal hospital stay greater than 5 days or an overnight readmission to hospital within 42 days of birth was significantly reduced for women who had a planned VBAC compared to ERCS only if they had any prior vaginal births. This apparent protective effect of prior vaginal delivery is consistent with several previous studies [39,46,47] and might be explained by women with a prior vaginal delivery having less complicated and possibly quicker labors that are more likely to result in a successful VBAC compared to women without a prior vaginal birth [16].

We also found evidence that the number of prior cesarean sections modifies the relationship between planned mode of delivery and the mother’s risk of having a postnatal hospital stay greater than 5 days, such that the risk of this outcome was significantly raised in women who had a planned VBAC compared to ERCS only if they had two or more prior cesarean sections. This might be explained by women planning VBAC following two or more prior cesareans experiencing more complications than those with just one prior cesarean, as suggested by a limited number of previous studies [48]. However, we did not find evidence that the effect of planned mode of delivery on the other adverse outcomes we considered varied according to the number of prior cesarean sections a woman had. Our findings may alternatively reflect the fact that, in our study, women who had a planned VBAC following two or more prior cesareans were less likely than those with just one prior cesarean to have a successful VBAC.

To the best of our knowledge, this is the first study to examine the effects of mode of delivery after previous cesarean on breastfeeding at 6–8 weeks postpartum, and only two previous studies conducted in the US have investigated the influence of mode of delivery after previous cesarean on breastfeeding initiation [49,50]. Consistent with our findings, both of these previous studies reported that women who had a successful VBAC were more likely to initiate breastfeeding than those delivering by ERCS were. Additionally, comparable to our findings, one of the studies [49] reported that women delivering by cesarean section after an unsuccessful VBAC were also more likely to initiate breastfeeding than women delivering by ERCS were. Our findings do not appear to be explained by differences in sociodemographic, maternal medical, and obstetric-related factors. Instead, they may reflect a greater intention to breastfeed, a higher level of breastfeeding self-efficacy, or a greater level of support to breastfeed among women who had a planned VBAC compared to ERCS.

In keeping with the findings of the AHRQ review [16], our study suggests that the risk of intrapartum stillbirth or neonatal death, excluding deaths from congenital abnormalities, is increased with planned VBAC compared to ERCS, although our absolute risk estimates are on the lower range of those reported by the review. This may reflect the fact that only two of the studies identified by the AHRQ review were population-based, and most of the studies did not exclude noncephalic births, which are associated with a higher risk of adverse outcome [51,52]. Furthermore, the recruitment period of the identified studies extends back to the 1990s. Findings from such studies may have limited relevance to current populations, owing to advances in obstetric and neonatal care and changes in clinical practice and population characteristics since this time. Having said that, our absolute and relative risk estimates for intrapartum stillbirth or neonatal death, excluding deaths from congenital abnormalities, are nevertheless within the range of those reported by a previous Scottish population-based study [53] included in the AHRQ review that was conducted in the 1990s, noting the large CIs reported in this earlier Scottish study (estimates 0.129%, 95% CI 0.079%–0.199% for planned VBAC versus 0.01%, 95% CI 0–0.061% for ERCS, aOR 11.7, 95% CI 1.4–101.6). Our study also adds to the limited evidence [16] about other measures of adverse perinatal outcome. Our findings are consistent with two large population-based studies conducted in the US [54,55] and several small non-population-based studies [36,37,56] that have reported an increased risk of various measures of neonatal morbidity or composite adverse neonatal outcome with planned VBAC compared to ERCS.

### Strengths and limitations

A major strength of our study is its large population-based design, which not only minimized the risk of selection bias but also maximized statistical power. We were also able to assess the effect of planned mode of delivery after previous cesarean on a comprehensive range of maternal and perinatal outcomes among women considered eligible to have a planned VBAC based on current UK guidelines. The inclusion of women in the ERCS group who are not eligible to have a planned VBAC has been highlighted as a key limitation with much of the existing literature [16]. Although we acknowledge that we lacked the data to exclude all women with contraindications to planned VBAC, in particular those with any prior classical cesarean scars or previous uterine rupture, we believe our study is likely to have excluded the vast majority of ineligible women. Indeed, a previous study suggests that only 2% of women giving birth after previous cesarean section in the UK have any previous non-low-transverse incisions [28]. Another strength of our study is that we were also able to examine outcomes according to whether planned VBAC was attempted with or without labor induction compared to ERCS, which few previous studies have examined despite the potential of labor induction to influence risk estimates [57]. A further strength of our study is that we were able to explore the influence of multiple a priori covariates on the associations studied, which many studies in this area have not done. However, as in other observational studies, we cannot rule out the possibility of residual confounding as an explanation for some or all of the differences we found. Although a large randomized controlled trial would be the gold-standard methodology for assessing the effects of planned mode of delivery after previous cesarean section, a previous study [37] provides strong evidence that such a trial is unlikely to be feasible in practice, as women are unlikely to consent to participate. As a consequence, large population-based observational studies such as ours offer the best opportunity to inform evidence in this area.

We recognize that the criteria we used to define planned mode of delivery could misclassify women who planned ERCS but went into labor before their scheduled delivery date. However, confining the analysis to births delivered at 39 or more weeks’ gestation, the gestation recommended by UK guidelines to carry out ERCS [8,9], resulted in little change in the effect estimates. This suggests our findings are robust to this potential misclassification. We also acknowledge that some misclassification of the other variables of interest may have occurred, which could have either biased the findings toward the null or under- or overestimated effects depending on whether any misclassification was random or systematic in nature. However, the completeness and quality of routinely collected Scottish data are considered to be very high, with some of the data sources containing statutorily collected data and many of the data sources undergoing regular quality assurance checks as outlined in the methods. The proportion of the study population that had missing data on one or more covariates is acknowledged as another limitation, although our use of multiple imputation is considered a valid approach for handing this issue, assuming the unobserved data are missing at random and the imputation models have been correctly specified [58]. Lastly, although all our analyses were prespecified based on clear hypotheses and biological plausibility, we acknowledge that the performance of multiple comparisons increased the risk of type 1 error.

### Conclusions and implications

This study suggests that among women considered eligible to have a planned VBAC, planned VBAC compared to ERCS is associated with an increased risk of the mother having serious birth-related maternal and perinatal complications. Conversely, our study suggests planned VBAC is associated with an increased likelihood of breastfeeding, whereas the effect on other maternal outcomes appears to differ according to whether a woman has any prior vaginal deliveries and the number of prior cesarean sections she has had. However, the absolute risk of adverse maternal and perinatal outcomes was found to be small for either delivery approach. The findings of this study are likely to be generalizable to other high-income countries with similar population characteristics and clinical practice. Although our findings can be used to manage and counsel women with previous cesarean section, as recommended by current guidelines, it is important to highlight that our study did not consider all possible outcomes. As noted in the latest Royal College of Obstetricians and Gynaecologists (RCOG) guidelines [8], there is a wealth of evidence to show that ERCS is associated with an increased risk of serious maternal morbidity in subsequent pregnancies, including morbidly adherent placenta. Such information should be considered alongside the evidence presented in this study. However, further research is needed to investigate other longer-term outcomes for women and their children associated with planned mode of delivery after previous cesarean section [8, 16].

## Supporting information

S1 RECORD Checklist RECORD, REporting of studies conducted using observational routinely-collected data. (DOCX)

S1 Text Extract from application to the Public Benefit and Privacy Panel for Health and Social Care Scotland, taken from application submitted in March 2018. (DOCX)

S1 Table Data sources, codes, and database fields used to identify study population, exposures, outcomes, and covariates. (DOCX)

S2 Table Complete case analysis of maternal and perinatal outcomes following planned VBAC compared to ERCS. ERCS, elective repeat cesarean section; VBAC, vaginal birth after previous cesarean.
(DOCX)

S3 Table Complete case analysis of maternal and perinatal outcomes following planned VBAC with and without labor induction compared to ERCS. ERCS, elective repeat cesarean section; VBAC, vaginal birth after previous cesarean.
(DOCX)

S4 Table Complete case analysis of maternal and perinatal outcomes following successful VBAC and in-labor nonelective repeat cesarean section compared to ERCS. ERCS, elective repeat cesarean section; VBAC, vaginal birth after previous cesarean.
(DOCX)

S5 Table Maternal and perinatal outcomes following planned VBAC compared to ERCS at ≥39 weeks’ gestation. ERCS, elective repeat cesarean section; VBAC, vaginal birth after previous cesarean.
(DOCX)

S6 Table Maternal and perinatal outcomes following planned VBAC with and without labor induction compared to ERCS at ≥39 weeks’ gestation. ERCS, elective repeat cesarean section; VBAC, vaginal birth after previous cesarean.
(DOCX)

S7 Table Maternal and perinatal outcomes following successful VBAC and in-labor nonelective repeat cesarean section compared to ERCS at ≥39 weeks’ gestation. ERCS, elective repeat cesarean section; VBAC, vaginal birth after previous cesarean.
(DOCX)

S8 Table SSBID identified intrapartum stillbirth or neonatal death excluding deaths from congenital abnormalities by planned and actual mode of delivery after previous cesarean section. SSBID, Scottish Stillbirth and Infant Death Survey.
(DOCX)

## Abbreviations

AHRQ: Agency for Healthcare Research and Quality
aOR: adjusted odds ratio
aRR: adjusted risk ratio
CHI: community health index number
CHSP-PS: Child Health Surveillance Programme Pre-School system
CI: confidence interval
ERCS: elective repeat cesarean section
ISD: Information Services Division
NC: not calculated because of low number of events
NHS: National Health Service
NIHR: National Institute for Health Research
NRES: National Research Ethics Service
NRS: National Records of Scotland
NS-SEC: National Statistics Socio-Economic Classification
OR: odds ratio
RCOG: Royal College of Obstetricians and Gynaecologists
RECORD: REporting of studies Conducted using Observational Routinely-collected Data
RR: risk ratio
SMR01: Scottish Morbidity Record General/Acute Inpatient and Day case dataset
SMR02: Scottish Morbidity Record Maternity Inpatient and Day Case dataset
SSBID: Scottish stillbirth and infant death survey
TOLAC: trial of labor after previous cesarean
VBAC: vaginal birth after previous cesarean
