# Supplementary material for: Planned mode of delivery after previous cesarean section and short-term maternal and perinatal outcomes: A population-based record linkage cohort study in Scotland
Source: PLoS Med. 2019 Sep 24;16(9):e1002913. doi: 10.1371/journal.pmed.1002913 (PMC6759152; doi:10.1371/journal.pmed.1002913)
Supplement: S1 Text — (DOCX) [file pmed.1002913.s002.docx]

**S1 Text. Extract from application to the Public Benefit and Privacy Panel for Health and Social Care Scotland, taken from application submitted in March 2018**

*We did not publish or pre-register an analysis plan, but a summary of the proposed study exposures, outcomes and statistical methods was included as part of the application to the Public Benefit and Privacy Panel for Health and Social Care Scotland to obtain the data. An extract of relevant text from this application is shown below. Please note this was submitted as part of a larger programme of research that intends to investigate the short and longer-term outcomes for women and their children according to planned mode of delivery after previous caesarean section. As such more data was requested than was included in this manuscript, and the below text reflects that of a data application as opposed to a formal pre-registered analysis plan.*

**Background**: Many countries, including the UK, have seen a rise in their caesarean section rate (now ~25% of all birhs in UK) leading to an increasing proportion of women embarking on a subsequent pregnancy with a history of previous caesarean section (estimated at ~100,000 women per year in UK). Broad policy consensus in high-income countries supports offering pregnant women who have had previous caesarean delivery a choice between planning to have another caesarean, known as an elective repeat caesarean section (ERCS), or attempting a vaginal delivery, known as a vaginal birth after previous caesarean (VBAC, also known as trial of labour). This is provided that they do not have contraindications to planned VBAC such as placenta praevia (low-lying placenta), where a caesarean section is clearly necessary. Current UK guidelines advise that such women should be counselled about the risks and benefits of ERCS compared to planned VBAC to help them make informed decisions with respect to this aspect of their maternity care. Furthermore, previous research suggests that many women would find it helpful to have access to accurate, comprehensive and well-balanced information about the risks and benefits of the different ways of giving birth after a previous caesarean section when making what many view as a very difficult decision. However, a number of significant limitations have been highlighted with the existing evidence, and there have been calls for methodologically rigorous studies to assess both the short and longer-term outcomes for women and their children of intended mode of delivery after prior caesarean section and thus fill this evidence gap.

-Objective 1a, to investigate the effect of elective repeat caesarean section (ERCS) compared to planned vaginal birth after caesarean section (VBAC) on the baby/child’s health outcomes and short-term health outcomes for the mother

**Study design**: Retrospective population-based cohort study.

**Participants/Eligibility criteria**: Include all births from 2002-2015 (cohort selection period) to women who have had one or more previous caesarean sections, identified from the Scottish Morbidity Record Maternity Inpatient and Day Case dataset (SMR02). Dependent on the data available and the data quality, the research team plan to try and exclude women with contraindications to planned VBAC based on current UK guidelines[1] (e.g. placenta praevia in latest pregnancy in cohort selection period or uterine rupture in any previous pregnancy).

**Methods**: The following linked data sources will be used to derive information on exposures, outcomes, potential cofounding, mediating or moderating factors and take account of censoring if appropriate for births meeting the eligibility criteria: SMR02 records from 1981-2015 (births in cohort selection period to eligible women and all their previous pregnancy records); Child Health Surveillance Programme Pre-School system (CHSP-PS) from 2002-2016; Scottish Stillbirth and Infant Death survey (SSBID) from 2002-2012; National Records of Scotland (NRS) infant deaths and deaths (for mother and child) from 2002-2016; Scottish Birth Record (SBR) from 2002-2016; Scottish Morbidity Record General/ Acute Inpatient and Day Case dataset (SMR01) from 1981-2016 for the mother and SMR01 from 2002-2016 for the child; Community Health Index (CHI) database from 2002-2016; and NRS live births and stillbirths from 1981-2015.

**Outcomes**: Maternal intrapartum and postpartum complications/morbidity (e.g. peripartum hysterectomy, uterine rupture, puerperal infections); Breastfeeding; intrapartum stillbirth or neonatal death excluding deaths from congenital anomalies; Admission to a neonatal unit, baby/child morbidity (e.g. infant respiratory morbidity, Apgar score, infection).

**Statistical analysis:** A descriptive analysis of the comparison groups, outcomes, potential confounders, mediators and moderators will first be conducted. Internal and external validation of the data will be performed by, for example, doing checks such as plausible birthweight for gestational age and comparing whether the prevalence of data items is consistent with published sources where available. Missing data and the factors associated with “missingness” will be assessed to determine the most appropriate method of addressing this in the analysis (e.g. complete case analysis, multiple imputation, inverse probability weighting). Standard statistical methods will be used to estimate the effect of **intended mode of delivery** on outcomes: logistic regression will be used to estimate odds ratios for rare binary outcomes (e.g. peripartum hysterectomy); modified Poisson regression will be used to estimate risk ratios for more common binary outcomes (e.g. breastfeeding); or Cox regression will be used to allow for follow-up time and take account of censoring if appropriate using information on deaths and emigration ascertained from the National Records of Scotland Deaths data and the CHI database respectively. In a series of secondary analyses, for comparative purpose, outcomes will also be examined according to **actual mode of delivery**. The influence of potential confounders, mediators and moderators on the association between intended mode of delivery and outcomes will be explored.

The main analyses will be confined to singleton births of cephalic presentation at term (≥37 weeks gestation). This is the main group of women current UK guidelines[1] recommend are candidates for and should be counselled about intended mode of delivery following previous caesarean section.

1. Royal College of Obstetricians and Gynaecologists. Birth After Previous Caesarean Birth, Green-top Guideline No. 45 London, 2015.
