# Supplementary material for: Planned mode of delivery after previous cesarean section and short-term maternal and perinatal outcomes: A population-based record linkage cohort study in Scotland
Source: PLoS Med. 2019 Sep 24;16(9):e1002913. doi: 10.1371/journal.pmed.1002913 (PMC6759152; doi:10.1371/journal.pmed.1002913)
Supplement: S1 Table — (DOCX) [file pmed.1002913.s003.docx]

**S1 Table. Data sources, codes and database fields used to identify study population, exposures, outcomes and covariates**

|  | **Data source** | **Database fields/codes** |
| --- | --- | --- |
| **Inclusion criteria** |  |  |
| ≥ 1 previous cesarean sections | SMR02 | *Previous caesarean sections* field ≥1 OR at least one previous delivery with a code for cesarean section in *mode of delivery* field (7 or 8) and/or an OPCS-4 or OPCS-3 code for cesarean section (R17-R18, R251, 764-766, 769) |
| Singleton birth | NRS live births and stillbirths | *Numbirths* field=1 |
| Term birth | SMR02 | Gestation at delivery 37-41 completed weeks according to *Estimated gestation* field (containing number of completed weeks of gestation as judged by the clinician, usually on the basis of ultrasound) OR according to gestation derived from *date of delivery* and *date of last menstrual period* fields if *Estimated gestation* missing (0.5% of eligible births) |
| **Exclusion criteria** |  |  |
| Non-cephalic presentation | SMR02 | Delivery episodes with a code for breech or shoulder in *presentation at delivery* field (4 or 6) OR a code for breech delivery or breech extraction in *mode of delivery* field (5 or 6) OR an ICD-10 code for breech delivery or breech extraction (O801, O830-O831) OR an OPCS-4 code for breech delivery or breech extraction (R19-R20) OR an ICD-10 code for maternal care for malpresentation of fetus (0320-0322, 0326-0329) |
| Placenta praevia | SMR02 | Delivery episodes with an ICD-10 code for placenta praevia (O440-O441) |
| Abdominal pregnancy | SMR02 | Delivery episodes with an ICD-10 code for delivery of or maternal care for viable fetus in abdominal pregnancy (O833, O367) |
| Known or suspected disproportion of maternal and/or fetal origin | SMR02 | Delivery episodes with an ICD-10 code for maternal care for known or suspected disproportion (O33) |
| Tumour of corpus uteri | SMR02 | Delivery episodes with an ICD-10 code for maternal care for tumour of corpus uteri (O341) |
| Pre-labor non-elective cesarean section | SMR02 | Code for non-elective cesarean section in *mode of delivery* field (8) AND *duration of labor* field=0 |
| Antepartum stillbirth | NRS stillbirths | Code for antepartum stillbirth in *period of death* field (1) |
| **Exposures** |  |  |
| Elective repeat cesarean section (ERCS) | SMR02 | Code for elective cesarean section in *mode of delivery* field (7) in women with ≥ 1 previous cesarean sections |
| Planned vaginal birth after previous cesarean section (Planned VBAC) | SMR02 | Code for vaginal birth in *mode of delivery* field (0, 1, 2, 3, 4, A, B, C, D or E) OR code for non-elective cesarean section in *mode of delivery* field (8) AND *duration of labour* field ≥1 hour in women with ≥1 previous cesarean sections |
| Planned VBAC without labor induction | SMR02 | Criteria for Planned VBAC AND code for none in *induction of labour* field (0) |
| Planned VBAC with labor induction | SMR02 | Criteria for Planned VBAC AND code for induction of labor using artificial rupture of membranes (ARM), oxytocics, ARM & oxytocics, prostaglandins, prostaglandins & ARM, prostaglandins & oxytocics, prostaglandins & ARM & oxytocics or other method in *induction of labour* field (1-8) |
| Successful vaginal birth after previous cesarean section (Successful VBAC) | SMR02 | Code for vaginal birth in *mode of delivery* field (0, 1, 2, 3, 4, A, B, C, D or E) in women with ≥ 1 previous cesarean sections |
| In-labor non-elective repeat cesarean section | SMR02 | Code for non-elective cesarean section in *mode of delivery* field (8) AND *duration of labour* field ≥1 hour in women with ≥1 previous cesarean sections |
| **Maternal outcomes** |  |  |
| Uterine rupture | SMR02 | Delivery episodes with an ICD-10 code for uterine rupture (O710-O711) |
| Peripartum hysterectomy | SMR02 and SMR01 | OPCS-4 code for hysterectomy (Q071-Q075, Q08, R251) within 6 weeks of delivery |
| Blood transfusion | SMR02 | Delivery episodes with an OPCS-4 code for blood transfusion (X331-X333, X337-X339, X341) |
| Puerperal sepsis | SMR02 and SMR01 | ICD-10 code for puerperal sepsis (O85) within 6 weeks of delivery |
| Other puerperal infection | SMR02 and SMR01 | ICD-10 code for other puerperal infections (O86) within 6 weeks of delivery |
| Surgical injury (Damage to bowel, bladder or ureter requiring surgical repair) | SMR02 and SMR01 | OPCS-4 code for any of the following within 6 weeks of delivery: total excision of colon and rectum, total excision of colon, extended excision of right hemicolon, other excision of right hemicolon, excision of transverse colon, excision of left hemicolon, excision of sigmoid colon, other excision of colon, exteriorisation of caecum, other exteriorisation of colon, subtotal excision of colon, exteriorisation of colon, repair of anus, other operations on the anal sphincter to control continence, excision of ureter, urinary diversion, replantation of ureter, other connection of ureter, repair of ureter, incision of ureter, other open operations on ureter,therapeutic nephroscopic operations on ureter, therapeutic ureteroscopic operations on ureter, other therapeutic endoscopic operations on ureter, percutaneous ureteric stent procedures, total excision of bladder, partial excision of bladder, enlargement of bladder, other repair of bladder or open drainage of bladder (H04-H11, H14-H15, H29, H32, H50, H57, M18-M23, M25-M27, M29, M33-M38) |
| Third or fourth degree perineal tear | SMR02 | Delivery episodes with a code for third- or fourth-degree tear in *tears* field (3 or 4) OR ICD-10 code for third- or fourth-degree perineal laceration during delivery (O702-0703) OR OPCS-4 code for repair of obstetric laceration of perineum and sphincter of anus or repair of obstetric laceration of perineum and sphincter and mucosa of anus (R322, R325) |
| Length of postnatal hospital stay | SMR02 and SMR01 | Derived from *date of delivery* and latest *date of* *discharge* of the continuous inpatient stay, with continuous inpatient stays identified as episodes where the difference between the discharge date of the episode and the admission date of the next episode is less than 0 OR is between 0-1 day AND either the discharged to/type of discharge of the episode is a transfer or the admission from/type of admission of the next episode is a transfer. |
| Overnight readmission to hospital within 42 days of giving birth | SMR02 and SMR01 | Women readmitted to hospital within 42 days of delivery, excluding planned transfers and readmissions of less than one day. Planned transfers identified as episodes where the difference between the admission date of the episode and the discharge date of the previous episode is less than 0 OR is between 0-1 day AND either the admission from/type of admission is a transfer or the discharged to/type of discharge of the previous episode is a transfer. Maternal deaths within the delivery episode were excluded, identified using discharge type 40-43 (death). Women with a length of postnatal hospital stay >42 days were also excluded. |
| Any breastfeeding at birth or hospital discharge | SMR02 | Code for breast only or mixed (breast and formula) in *first feed given* (immediately following delivery) or *feed on discharge* fields (1 or 4) |
| Exclusive breastfeeding at 6-8 week review | CHSP-PS | Method of feeding at 6-8 week review breast milk only |
| Any breastfeeding at 6-8 week review | CHSP-PS | Method of feeding at 6-8 week review breast milk only or mixed breast and formula milk |
| **Perinatal outcomes** |  |  |
| Intrapartum stillbirth or neonatal mortality | NRS deaths or SSBID in sensitivity analysis | Code for intrapartum stillbirth or death within four weeks of birth in *period of death* field (2-6). Deaths from congenital anomalies were excluded, identified from NRS deaths data as deaths with a primary cause of death ICD-10 code for congenital malformations, deformations and chromosomal abnormalities (Q00-Q99) and in SSBID as deaths with a paediatric classification code for congenital anomaly (1-7) |
| Admission to a neonatal unit | SMR02 | *Neonatal indicator* field. A neonatal unit defined by ISD as a nursery in which accommodation, equipment, staffing, spacing, temperature, and other support facilities are such as to permit the care of babies who require a level of care above that associated with healthy babies. |
| Resuscitation requiring drugs and/or intubation | SMR02 | Code for bag and mask with drugs, intubation for IPVV with/without drugs or drugs only in *resuscitation* field (3-6) |
| Apgar score at 5 minutes | SMR02 | *Apgar score* field |
| **Socio-demographic covariates** |  |  |
| Maternal age | SMR02 | Derived from mother’s *date of birth* and *date of delivery* in current pregnancy |
| Mother’s country of birth | NRS live and stillbirths | *Mother’s Country of Birth* field |
| Marital status/registration type | NRS live and stillbirths | *Parents married indicator* field |
| Socio-economic status | NRS live and stillbirths | Socio-economic status of mother if sole registered birth or highest of mother or father’s socio-economic status for births registered inside marriage or jointly registered by both parents outside marriage. Socio-economic status defined by National Statistics Socio-Economic Classification (NS-SEC) based on occupation and employment status |
| **Maternal medical and pregnancy-related covariates** |  |  |
| Number of previous cesarean sections | SMR02 | *Previous caesarean sections* field. Number of previous cesarean sections according to *previous caesarean sections* field was cross-checked against woman’s previous delivery records in SMR02. Where this was found to be less than the number of previous cesarean sections observed to date (14% of eligible births), it was overwritten with the higher number. Also, if the previous caesarean sections field was missing (0.2% of eligible births), number of previous cesarean sections was derived from the number of previous cesarean sections observed to date. |
| Any prior vaginal delivery | SMR02 | Derived from parity (number of previous pregnancies resulting in either a live birth or stillbirth) and number of previous cesarean sections. Woman’s previous delivery records were also examined for evidence of any prior vaginal deliveries. |
| Inter-pregnancy interval | SMR02 | Derived from interval between *date of delivery* of current pregnancy and *date of delivery* of previous delivery minus gestational age at delivery of current pregnancy |
| Smoking status at booking | SMR02 | *Booking smoking history* field |
| Maternal BMI at booking | SMR02 | Derived from *height* and *weight of mother at booking* fields. Maternal height values < 120 cm and > 200 cm, weight of mother at booking values < 32kg and > 180 kg and maternal BMI at booking values ≤15 and >80 kg/m2 were considered to be implausible based on published values[1,2]. If height was missing or implausible, it was set to the median value observed in the woman’s other records in SMR02. Implausible values of maternal weight or BMI at booking were set to missing. |
| Hypertensive disorder | SMR02 | Antenatal or delivery episodes with an ICD-10 code for pre-existing or gestational hypertensive disorder (O10-O11, O13-O16, I10) |
| Diabetes mellitus | SMR02 | Antenatal or delivery episodes with a code for pre-existing or gestational diabetes mellitus in *diabetes* field (1-3) OR an ICD-10 code for pre-existing or gestational diabetes mellitus (O24, E10-E11) |
| Pre-labor rupture of membranes | SMR02 | Antenatal or delivery episodes with an ICD-10 code for premature rupture of membranes (O42) |
| **Infant-related covariates** |  |  |
| Gender of child | NRS live births and stillbirths | *Sex (gender)* field |
| Gestational age at delivery | SMR02 | *Estimated gestation* field (containing number of completed weeks of gestation as judged by the clinician, usually on the basis of ultrasound) or if missing (0.5% of eligible births) derived from *date of delivery* and *date of last menstrual period* fields |
| Birth weight centile | SMR02 | Derived from gestational age at delivery*,* birthweight and gender of child using sex-specific birth weight for gestational age centiles as reported by Bonellie et at[3]. Implausible birth weights for gestational age were identified as those more than twice the inter-quartile range below and above the first and third quartile, respectively, using the sex-specific birth weight for gestational age centiles reported by Bonellie et at[3]. Implausible values were set to missing. |

**References**

1. Knight M, Kurinczuk JJ, Spark P, Brocklehurst P, System UKOS. Extreme obesity in pregnancy in the United Kingdom. Obstet Gynecol. 2010;115(5):989-97. Epub 2010/04/23. doi: 10.1097/AOG.0b013e3181da8f09. PubMed PMID: 20410773.

2. Scotland ISD. Data dictionary SMR02 Maternity Inpatient and Day Case. Available from: <https://www.ndc.scot.nhs.uk/Data-Dictionary/SMR-Datasets/SMR02-Maternity-Inpatient-and-Day-Case/>.

3. Bonellie S, Chalmers J, Gray R, Greer I, Jarvis S, Williams C. Centile charts for birthweight for gestational age for Scottish singleton births. BMC Pregnancy Childbirth. 2008;8:5. Epub 2008/02/27. doi: 10.1186/1471-2393-8-5. PubMed PMID: 18298810; PubMed Central PMCID: PMCPMC2268653.
