# Supplementary material for: Planned mode of delivery after previous cesarean section and short-term maternal and perinatal outcomes: A population-based record linkage cohort study in Scotland
Source: PLoS Med. 2019 Sep 24;16(9):e1002913. doi: 10.1371/journal.pmed.1002913 (PMC6759152; doi:10.1371/journal.pmed.1002913)
Supplement: S2 Table — ERCS, elective repeat cesarean section; VBAC, vaginal birth after previous cesarean. (DOCX) [file pmed.1002913.s004.docx]

**S2 Table. Complete case analysis of maternal and perinatal outcomes following planned VBAC compared to ERCS**

|  | **Base model^1^ relative risk (95% CI)** | **Model A^2^ relative risk (95% CI)** | **Model B^3^ relative risk (95% CI)** | **Model C^4^ relative risk (95% CI)** |
| --- | --- | --- | --- | --- |
| ***Maternal outcomes*** |  |  |  |  |
| Uterine rupture | **6.26 (3.64-10.76)**  **P<0.001** | **6.28 (3.62-10.89)**  **P<0.001** | NC | - |
| Peripartum hysterectomy | 0.47 (0.19-1.16)  P=0.102 | NC | NC | - |
| Blood transfusion† | **2.12 (1.78-2.52)**  **P<0.001** | **2.09 (1.76-2.49)**  **P<0.001** | **2.74 (2.08-3.60)**  **P<0.001** | - |
| Puerperal sepsis‡¥ | **1.73 (1.25-2.38)**  **P=0.001** | **1.69 (1.22-2.33)**  **P=0.001** | 1.22 (0.73-2.01)  P=0.447 | - |
| Other puerperal infection‡¥ | 1.00 (0.90-1.11)  P=0.986 | 0.99 (0.89-1.10)  P=0.855 | 1.00 (0.85-1.18)  P=0.994 | - |
| Surgical injury | **1.96 (1.29-2.98)**  **P=0.002** | **2.01 (1.33-3.05)**  **P=0.001** | NC | - |
| Length of postnatal hospital stay >5 days†‡¥ | **0.88 (0.81-0.97)**  **P=0.007** | **0.89 (0.82-0.98)**  **P=0.013** | 0.92 (0.79-1.06)  P=0.252 | - |
| Readmission to hospital within 42 days of birth†‡¥ | **0.88 (0.80-0.96)**  **P=0.006** | **0.87 (0.79-0.96)**  **P=0.004** | 1.01 (0.88-1.16)  P=0.838 | - |
| Any breastfeeding at birth or hospital discharge | **1.18 (1.16-1.20)**  **P<0.001** | **1.19 (1.18-1.21)**  **P<0.001** | **1.18 (1.16-1.20)**  **P<0.001** | **1.15 (1.13-1.17)**  **P<0.001** |
| Exclusive breastfeeding at 6-8 week review | **1.34 (1.31-1.38)**  **P<0.001** | **1.37 (1.34-1.41)**  **P<0.001** | **1.38 (1.33-1.42)**  **P<0.001** | **1.31 (1.26-1.35)**  **P<0.001** |
| Any breastfeeding at 6-8 week review | **1.27 (1.24-1.30)**  **P<0.001** | **1.29 (1.26-1.31)**  **P<0.001** | **1.29 (1.26-1.33)**  **P<0.001** | **1.24 (1.21-1.27)**  **P<0.001** |
| ***Perinatal outcomes*** |  |  |  |  |
| Adverse perinatal outcome^a^†‡¥ | **1.22 (1.15-1.30)**  **P<0.001** | **1.22 (1.15-1.30)**  **P<0.001** | **1.37 (1.25-1.50)**  **P<0.001** | **1.59 (1.45-1.75)**  **P<0.001** |
| Intrapartum stillbirth or neonatal death | **6.59 (2.43-17.87)**  **P<0.001** | NC | NC | NC |
| Admitted to a neonatal unit†‡¥ | 1.00 (0.93-1.07)  P=0.967 | 1.00 (0.94-1.07)  P=0.960 | **1.13 (1.02-1.25)**  **P=0.015** | **1.33 (1.20-1.47)**  **P<0.001** |
| Resuscitation requiring drugs and/or intubation†‡¥ | **4.67 (3.84-5.68)**  **P<0.001** | **4.68 (3.84-5.70)**  **P<0.001** | **4.74 (3.48-6.45)**  **P<0.001** | **4.71 (3.48-6.37)**  **P<0.001** |
| Apgar score <7 at 5 minutes†‡¥ | **3.45 (2.90-4.09)**  **P<0.001** | **3.46 (2.91-4.11)**  **P<0.001** | **3.37 (2.63-4.32)**  **P<0.001** | **3.73 (2.90-4.80)**  **P<0.001** |

1 Base model adjusted for year of delivery.

2 Model A adjusted for year of delivery and socio-demographic factors (maternal age, mother’s country of birth, marital status/registration type and socio-economic status).

3 Model B adjusted for variables in Model A and additionally adjusted for maternal medical and pregnancy-related factors (number of previous cesarean sections, any prior vaginal delivery, inter-pregnancy interval, maternal smoking status at booking, maternal BMI at booking, hypertensive disorder where † is shown, diabetes where ‡ is shown and pre-labor rupture of membranes where ¥ is shown).

4 Model C adjusted for variables in Model B and additionally adjusted for infant-related factors (sex of infant, gestational age at delivery and birth weight centile).

^a^ Includes intrapartum stillbirth or neonatal death, admission to a neonatal unit, resuscitation requiring drugs and/or intubation or an Apgar score <7 at 5 minutes.

NC – not calculated because of low number of events.

Bold text indicates statistically significant findings at the 5% level.
