# Supplementary material for: Planned mode of delivery after previous cesarean section and short-term maternal and perinatal outcomes: A population-based record linkage cohort study in Scotland
Source: PLoS Med. 2019 Sep 24;16(9):e1002913. doi: 10.1371/journal.pmed.1002913 (PMC6759152; doi:10.1371/journal.pmed.1002913)
Supplement: S3 Table — ERCS, elective repeat cesarean section; VBAC, vaginal birth after previous cesarean. (DOCX) [file pmed.1002913.s005.docx]

**S3 Table. Complete case analysis of maternal and perinatal outcomes following planned VBAC with and without labor induction compared to ERCS**

|  | **Planned VBAC without labor induction vs. ERCS** | | | |  | **Planned VBAC with labor induction vs. ERCS** | | | |
| --- | --- | --- | --- | --- | --- | --- | --- | --- | --- |
|  | **Base model^1^ relative risk (95% CI)** | **Model A^2^ relative risk (95% CI)** | **Model B^3^ relative risk (95% CI)** | **Model C^4^ relative risk (95% CI)** |  | **Base model^1^ relative risk (95% CI)** | **Model A^2^ relative risk (95% CI)** | **Model B^3^ relative risk (95% CI)** | **Model C^4^ relative risk (95% CI)** |
| ***Maternal outcomes*** |  |  |  |  |  |  |  |  |  |
| Uterine rupture | **5.92**  **(3.38-10.37)**  **P<0.001** | **5.89**  **(3.34-10.41)**  **P<0.001** | NC | - |  | **7.89**  **(3.99-15.61)**  **P<0.001** | **8.17**  **(4.08-16.35)**  **P<0.001** | NC | - |
| Peripartum hysterectomy | 0.39  (0.13-1.12)  P=0.080 | NC | NC | - |  | 0.85  (0.20-3.64)  P=0.828 | NC | NC | - |
| Blood transfusion† | **2.06**  **(1.72-2.47)**  **P<0.001** | **2.04**  **(1.70-2.45)**  **P<0.001** | **2.59**  **(1.95-3.44)**  **P<0.001** | - |  | **2.42**  **(1.83-3.19)**  **P<0.001** | **2.38**  **(1.81-3.14)**  **P<0.001** | **3.70**  **(2.42-5.68)**  **P<0.001** | - |
| Puerperal sepsis‡¥ | **1.51**  **(1.06-2.14)**  **P=0.021** | **1.48**  **(1.04-2.10)**  **P=0.028** | 1.06  (0.63-1.81)  P=0.821 | - |  | **2.55**  **(1.56-4.16)**  **P<0.001** | **2.49**  **(1.53-4.06)**  **P<0.001** | 2.0  (0.90-4.49)  P=0.091 | - |
| Other puerperal infection‡¥ | 0.98  (0.88-1.10)  P=0.757 | 0.97  (0.87-1.09)  P=0.639 | 0.97  (0.82-1.16)  P=0.759 | - |  | 1.10  (0.91-1.32)  P=0.325 | 1.08  (0.90-1.30)  P=0.415 | 1.16  (0.86-1.56)  P=0.326 | - |
| Surgical injury | **2.12**  **(1.38-3.25)**  **P<0.001** | **2.17**  **(1.42-3.32)**  **P<0.001** | NC | - |  | 1.33  (0.56-3.15)  P=0.518 | 1.37  (0.58-3.26)  P=0.470 | NC | - |
| Length of postnatal hospital stay >5 days†‡¥ | **0.83**  **(0.76-0.92)**  **P<0.001** | **0.84**  **(0.77-0.93)**  **P=0.001** | 0.88  (0.75-1.03)  P=0.118 | - |  | 1.11  (0.96-1.30)  P=0.167 | 1.11  (0.95-1.29)  P=0.189 | 1.08  (0.83-1.40)  P=0.554 | - |
| Readmission to hospital within 42 days of birth†‡¥ | **0.81**  **(0.73-0.89)**  **P<0.001** | **0.80**  **(0.72-0.89)**  **P<0.001** | 0.92  (0.79-1.06)  P=0.246 | - |  | **1.19**  **(1.01-1.39)**  **P=0.036** | **1.18**  **(1.00-1.38)**  **P=0.045** | **1.51**  **(1.21-1.89)**  **P<0.001** | - |
| Any breastfeeding at birth or hospital discharge | **1.19**  **(1.17-1.20)**  **P<0.001** | **1.19**  **(1.17-1.21)**  **P<0.001** | **1.18**  **(1.16-1.20)**  **P<0.001** | **1.15**  **(1.13-1.18)**  **P<0.001** |  | **1.15**  **(1.12-1.18)**  **P<0.001** | **1.20**  **(1.17-1.23)**  **P<0.001** | **1.17**  **(1.13-1.21)**  **P<0.001** | **1.12**  **(1.08-1.16)**  **P<0.001** |
| Exclusive breastfeeding at 6-8 week review | **1.38**  **(1.34-1.42)**  **P<0.001** | **1.39**  **(1.36-1.43)**  **P<0.001** | **1.39**  **(1.34-1.44)**  **P<0.001** | **1.32**  **(1.28-1.37)**  **P<0.001** |  | **1.20**  **(1.14-1.26)**  **P<0.001** | **1.29**  **(1.24-1.35)**  **P<0.001** | **1.31**  **(1.22-1.40)**  **P<0.001** | **1.20**  **(1.12-1.29)**  **P<0.001** |
| Any breastfeeding at 6-8 week review | **1.29**  **(1.26-1.32)**  **P<0.001** | **1.29**  **(1.27-1.32)**  **P<0.001** | **1.29**  **(1.36-1.33)**  **P<0.001** | **1.25**  **(1.21-1.28)**  **P<0.001** |  | **1.17**  **(1.13-1.22)**  **P<0.001** | **1.25**  **(1.21-1.30)**  **P<0.001** | **1.30**  **(1.23-1.37)**  **P<0.001** | **1.21**  **(1.15-1.28)**  **P<0.001** |

**S3 Table continued**

|  | **Planned VBAC without labor induction vs. ERCS** | | | |  | **Planned VBAC with labor induction vs. ERCS** | | | |
| --- | --- | --- | --- | --- | --- | --- | --- | --- | --- |
|  | **Base model^1^  relative risk (95% CI)** | **Model A^2^ relative risk (95% CI)** | **Model B^3^ relative risk (95% CI)** | **Model C^4^ relative risk (95% CI)** |  | **Base model^1^  relative risk (95% CI)** | **Model A^2^ relative risk (95% CI)** | **Model B^3^ relative risk (95% CI)** | **Model C^4^ relative risk (95% CI)** |
| ***Perinatal outcomes*** |  |  |  |  |  |  |  |  |  |
| Adverse perinatal outcome^a^†‡¥ | **1.15**  **(1.08-1.23)**  **P<0.001** | **1.16**  **(1.08-1.24)**  **P<0.001** | **1.33**  **(1.20-1.46)**  **P<0.001** | **1.52**  **(1.38-1.68)**  **P<0.001** |  | **1.51**  **(1.36-1.68)**  **P<0.001** | **1.49**  **(1.34-1.66)**  **P<0.001** | **1.58**  **(1.34-1.87)**  **P<0.001** | **2.03**  **(1.71-2.40)**  **P<0.001** |
| Intrapartum stillbirth or neonatal death | **6.9**  **(2.48-19.19)**  **P<0.001** | NC | NC | NC |  | **5.35**  **(1.29-22.12)**  **P=0.021** | NC | NC | NC |
| Admitted to a neonatal unit†‡¥ | 0.94  (0.88-1.02)  P=0.126 | 0.95  (0.88-1.02)  P=0.182 | **1.11**  **(1.00-1.24)**  **P=0.049** | **1.28**  **(1.15-1.42)**  **P<0.001** |  | **1.23**  **(1.09-1.39)**  **P<0.001** | **1.21**  **(1.08-1.37)**  **P=0.002** | **1.24**  **(1.03-1.49)**  **P=0.023** | **1.60**  **(1.32-1.94)**  **P<0.001** |
| Resuscitation requiring drugs and/or intubation†‡¥ | **4.69**  **(3.83-5.75)**  **P<0.001** | **4.72**  **(3.85-5.78)**  **P<0.001** | **4.50**  **(3.28-6.16)**  **P<0.001** | **4.53**  **(3.33-6.16)**  **P<0.001** |  | **5.66**  **(4.32-7.40)**  **P<0.001** | **5.59**  **(4.27-7.33)**  **P<0.001** | **6.13**  **(3.93-9.58)**  **P<0.001** | **6.07**  **(3.85-9.58)**  **P<0.001** |
| Apgar score <7 at 5 minutes†‡¥ | **3.24**  **(2.70-3.88)**  **P<0.001** | **3.26**  **(2.72-3.91)**  **P<0.001** | 3**.26**  **(2.52-4.21)**  **P<0.001** | **3.57**  **(2.75-4.63)**  **P<0.001** |  | **4.39**  **(3.43-6.62)**  **P<0.001** | **4.31**  **(3.37-5.53)**  **P<0.001** | **4.03**  **(2.77-5.86)**  **P<0.001** | **4.77**  **(3.24-7.01)**  **P<0.001** |

1 Base model adjusted for year of delivery.

2 Model A adjusted for year of delivery and socio-demographic factors (maternal age, mother’s country of birth, marital status/registration type and socio-economic status).

3 Model B adjusted for variables in Model A and additionally adjusted for maternal medical and pregnancy-related factors (number of previous cesarean sections, any prior vaginal delivery, inter-pregnancy interval, maternal smoking status at booking, maternal BMI at booking, hypertensive disorder where † is shown, diabetes where ‡ is shown and pre-labor rupture of membranes where ¥ is shown).

4 Model C adjusted for variables in Model B and additionally adjusted for infant-related factors (sex of infant, gestational age at delivery and birth weight centile).

^a^ Includes intrapartum stillbirth or neonatal death, admission to a neonatal unit, resuscitation requiring drugs and/or intubation or an Apgar score <7 at 5 minutes.

NC – not calculated because of low number of events

Bold text indicates statistically significant findings at the 5% level.
