# Supplementary material for: Planned mode of delivery after previous cesarean section and short-term maternal and perinatal outcomes: A population-based record linkage cohort study in Scotland
Source: PLoS Med. 2019 Sep 24;16(9):e1002913. doi: 10.1371/journal.pmed.1002913 (PMC6759152; doi:10.1371/journal.pmed.1002913)
Supplement: S4 Table — ERCS, elective repeat cesarean section; VBAC, vaginal birth after previous cesarean. (DOCX) [file pmed.1002913.s006.docx]

**S4 Table. Complete case analysis of maternal and perinatal outcomes following successful VBAC and in-labor non-elective repeat cesarean section compared to ERCS**

|  | **Successful VBAC vs. ERCS** | | | |  | **In-labor non-elective repeat cesarean section vs. ERCS** | | | |
| --- | --- | --- | --- | --- | --- | --- | --- | --- | --- |
|  | **Base model^1^  relative risk (95% CI)** | **Model A^2^ relative risk (95% CI)** | **Model B^3^ relative risk (95% CI)** | **Model C^4^ relative risk (95% CI)** |  | **Base model^1^  relative risk (95% CI)** | **Model A^2^ relative risk (95% CI)** | **Model B^3^ relative risk (95% CI)** | **Model C^4^ relative risk (95% CI)** |
| ***Maternal outcomes*** |  |  |  |  |  |  |  |  |  |
| Uterine rupture | 1.14  (0.51-2.56)  P=0.744 | 1.16  (0.51-2.60)  P=0.727 | NC | - |  | **19.28**  **(11.09-33.53)**  **P<0.001** | **19.28**  **(10.97-33.88)**  **P<0.001** | NC | - |
| Peripartum hysterectomy | 0.33  (0.10-1.10)  P=0.071 | NC | NC | - |  | 0.82  (0.24-2.76)  P=0.752 | NC | NC | - |
| Blood transfusion† | **1.95**  **(1.61-2.36)**  **P<0.001** | **1.93**  **(1.59-2.34)**  **P<0.001** | **2.37**  **(1.74-3.22)**  **P<0.001** | - |  | **2.54**  **(2.02-3.21**)  **P<0.001** | **2.51**  **(1.99-3.17**)  **P<0.001** | **3.60**  **(2.56-5.07**)  **P<0.001** | - |
| Puerperal sepsis‡¥ | 1.17  (0.79-1.73)  P=0.430 | 1.15  (0.78-1.70)  P=0.489 | 0.99  (0.55-1.78)  P=0.968 | - |  | **3.14**  **(2.12-4.66)**  **P<0.001** | **3.08**  **(2.08-4.57)**  **P<0.001** | 1.84  (0.95-3.57)  P=0.071 | - |
| Other puerperal infection‡¥ | **0.66**  **(0.58-0.75**)  **P<0.001** | **0.65**  **(0.57-0.74**)  **P<0.001** | **0.63**  **(0.51-0.77**)  **P<0.001** | - |  | **1.88**  **(1.65-2.13**)  **P<0.001** | **1.87**  **(1.65-2.13**)  **P<0.001** | **1.99**  **(1.64-2.41**)  **P<0.001** | - |
| Surgical injury | **0.11**  **(0.03-0.47**)  **P=0.003** | **0.12**  **(0.03-0.48**)  **P=0.003** | NC | - |  | **6.67**  **(4.37-10.18)**  **P<0.001** | **6.92**  **(4.56-10.49)**  **P<0.001** | NC | - |
| Length of postnatal hospital stay >5 days†‡¥ | **0.60**  **(0.53-0.67**)  **P<0.001** | **0.60**  **(0.53-0.67**)  **P<0.001** | **0.64**  **(0.54-0.76**)  **P<0.001** | - |  | **1.63**  **(1.46-1.82**)  **P<0.001** | **1.67**  **(1.50-1.87**)  **P<0.001** | **1.73**  **(1.44-2.08**) **P<0.001** | - |
| Readmission to hospital within 42 days of birth†‡¥ | **0.82**  **(0.74-0.92**)  **P<0.001** | **0.82**  **(0.73-0.91**) **P<0.001** | 0.95  (0.81-1.11)  P=0.495 | - |  | 1.02  (0.88-1.17)  P=0.835 | 1.01  (0.88-1.16)  P=0.899 | 1.20  (0.98-1.48)  P=0.078 | - |
| Any breastfeeding at birth or hospital discharge | **1.17**  **(1.15-1.19**)  **P<0.001** | **1.19**  **(1.17-1.21**)  **P<0.001** | **1.19**  **(1.17-1.21**)  **P<0.001** | **1.16**  **(1.14-1.19**)  **P<0.001** |  | **1.21**  **(1.19-1.24**)  **P<0.001** | **1.20**  **(1.18-1.22**)  **P<0.001** | **1.15**  **(1.12-1.18**)  **P<0.001** | **1.12**  **(1.09-1.15**)  **P<0.001** |
| Exclusive breastfeeding at 6-8 week review | **1.34**  **(1.30-1.38**) **P<0.001** | **1.40**  **(1.36-1.44**) **P<0.001** | **1.40**  **(1.35-1.45**) **P<0.001** | **1.33**  **(1.28-1.38**) **P<0.001** |  | **1.34**  **(1.29-1.39**) **P<0.001** | **1.32**  **(1.27-1.37**) **P<0.001** | **1.31**  **(1.25-1.38**) **P<0.001** | **1.25**  **(1.18-1.31**) **P<0.001** |
| Any breastfeeding at 6-8 week review | **1.25**  **(1.22-1.28**) **P<0.001** | **1.29**  **(1.26-1.32**) **P<0.001** | **1.31**  **(1.27-1.35**) **P<0.001** | **1.26**  **(1.22-1.30**) **P<0.001** |  | **1.31**  **(1.27-1.35**) **P<0.001** | **1.27**  **(1.24-1.31**) **P<0.001** | **1.24**  **(1.19-1.30**) **P<0.001** | **1.19**  **(1.15-1.24**) **P<0.001** |

**S4 Table continued**

|  | **Successful VBAC vs. ERCS** | | | |  | **In-labor non-elective repeat cesarean section vs. ERCS** | | | |
| --- | --- | --- | --- | --- | --- | --- | --- | --- | --- |
|  | **Base model^1^ relative risk (95% CI)** | **Model A^2^ relative risk (95% CI)** | **Model B^3^ relative risk (95% CI)** | **Model C^4^ relative risk (95% CI)** |  | **Base model^1^ relative risk (95% CI)** | **Model A^2^ relative risk (95% CI)** | **Model B^3^ relative risk (95% CI)** | **Model C^4^ relative risk (95% CI)** |
| ***Perinatal outcomes*** |  |  |  |  |  |  |  |  |  |
| Adverse perinatal outcome^a^†‡¥ | 1.07  (1.00-1.15)  P=0.065 | 1.06  (0.99-1.14)  P=0.085 | **1.23**  **(1.11-1.36**)  **P<0.001** | **1.45**  **(1.30-1.61**)  **P<0.001** |  | **1.61**  **(1.48-1.76**)  **P<0.001** | **1.65**  **(1.51-1.80**)  **P<0.001** | **1.76**  **(1.54-2.02**)  **P<0.001** | **1.98**  **(1.73-2.26**)  **P<0.001** |
| Intrapartum stillbirth or neonatal death | **4.60**  **(1.55-13.64**)  **P=0.006** | NC | NC | NC |  | **11.63**  **(3.89-34.75**)  **P<0.001** | NC | NC | NC |
| Admitted to a neonatal unit†‡¥ | **0.85**  **(0.79-0.92)**  **P<0.001** | **0.85**  **(0.78-0.92) P<0.001** | 1.01  (0.90-1.13)  P=0.926 | **1.19**  **(1.06-1.34**)  **P=0.003** |  | **1.38**  **(1.26-1.52**)  **P<0.001** | **1.42**  **(1.29-1.56**) **P<0.001** | **1.49**  **(1.29-1.73**) **P<0.001** | **1.68**  **(1.45-1.94**) **P<0.001** |
| Resuscitation requiring drugs and/or intubation†‡¥ | **4.62**  **(3.76-5.68) P<0.001** | **4.61**  **(3.74-5.67) P<0.001** | **4.37**  **(3.13-6.10) P<0.001** | **4.32**  **(3.11-6.00**) **P<0.001** |  | **5.58**  **(4.39-7.09) P<0.001** | **5.70**  **(4.48-7.26) P<0.001** | **5.76**  **(3.95-8.40) P<0.001** | **5.72**  **(3.94-8.29) P<0.001** |
| Apgar score <7 at 5 minutes†‡¥ | **2.76**  **(2.28-3.33**) **P<0.001** | **2.74**  **(2.26-3.32**) **P<0.001** | **2.55**  **(1.94-3.35**) **P<0.001** | **2.83**  **(2.14-3.73**) **P<0.001** |  | **5.22**  **(4.24-6.42**) **P<0.001** | **5.36**  **(4.35-6.60**) **P<0.001** | **5.41**  **(4.05-7.23**) **P<0.001** | **5.80**  **(4.33-7.77**) **P<0.001** |

1 Base model adjusted for year of delivery.

2 Model A adjusted for year of delivery and socio-demographic factors (maternal age, mother’s country of birth, marital status/registration type and socio-economic status).

3 Model B adjusted for variables in Model A and additionally adjusted for maternal medical and pregnancy-related factors (number of previous cesarean sections, any prior vaginal delivery, inter-pregnancy interval, maternal smoking status at booking, maternal BMI at booking, hypertensive disorder where † is shown, diabetes where ‡ is shown and pre-labor rupture of membranes where ¥ is shown).

4 Model C adjusted for variables in Model B and additionally adjusted for infant-related factors (sex of infant, gestational age at delivery and birth weight centile).

^a^ Includes intrapartum stillbirth or neonatal death, admission to a neonatal unit, resuscitation requiring drugs and/or intubation or an Apgar score <7 at 5 minutes.

NC – not calculated because of low number of events.

Bold text indicates statistically significant findings at the 5% level.
