# Supplementary material for: Planned mode of delivery after previous cesarean section and short-term maternal and perinatal outcomes: A population-based record linkage cohort study in Scotland
Source: PLoS Med. 2019 Sep 24;16(9):e1002913. doi: 10.1371/journal.pmed.1002913 (PMC6759152; doi:10.1371/journal.pmed.1002913)
Supplement: S5 Table — ERCS, elective repeat cesarean section; VBAC, vaginal birth after previous cesarean. (DOCX) [file pmed.1002913.s007.docx]

**S5 Table. Maternal and perinatal outcomes following planned VBAC compared to ERCS at ≥ 39 weeks gestation**

|  | **ERCS n outcome events/total n (%)** | **Planned VBAC n outcome events/total n (%)** | **Base model^1^ relative risk (95% CI)** | **Model A^2^ relative risk (95% CI)** | **Model B^3^ relative risk (95% CI)** | **Model C^4^ relative risk (95% CI)** |
| --- | --- | --- | --- | --- | --- | --- |
| ***Maternal outcomes*** |  |  |  |  |  |  |
| Uterine rupture | 16/31,880 (0.05) | 57/22,758 (0.25) | **4.65 (2.61-8.29) P<0.001** | **4.69 (2.61-8.43) P<0.001** | **5.95 (2.94-12.04) P<0.001** | - |
| Peripartum hysterectomy | 9/31,880 (0.03) | # (0.02) | 0.49 (0.15-1.61) P=0.244 | NC | NC | - |
| Blood transfusion† | 149/31,880 (0.47) | 268/22,758 (1.18) | **2.23 (1.81-2.75) P<0.001** | **2.19 (1.78-2.69) P<0.001** | **2.39 (1.90-3.01) P<0.001** | - |
| Puerperal sepsis‡¥ | 48/31,880 (0.15) | 57/22,758 (0.25) | **1.96 (1.32-2.89) P<0.001** | **1.91 (1.29-2.82) P=0.001** | **1.91 (1.21-2.99) P=0.005** | - |
| Other puerperal infection‡¥ | 660/31,880 (2.07) | 540/22,758 (2.37) | 1.09 (0.97-1.23) P=0.143 | 1.08 (0.96-1.22) P=0.193 | **1.30 (1.14-1.49) P<0.001** | - |
| Surgical injury | 24/31,880 (0.08) | # (0.17) | **2.41 (1.45-4.00) P<0.001** | **2.44 (1.47-4.03) P<0.001** | NC | - |
| Length of postnatal hospital stay >5 days†‡¥ | 729/31,880 (2.29) | 620/22,758 (2.72) | 1.02 (0.91-1.14) P=0.761 | 1.03 (0.92-1.15) P=0.644 | 1.11 (0.97-1.26) P=0.124 | - |
| Readmission to hospital within 42 days of birth^a^†‡¥ | 822/31,879 (2.58) | 554/22,757 (2.43) | 0.97 (0.86-1.08) P=0.553 | 0.96 (0.86-1.08) P=0.528 | 1.04 (0.92-1.18) P=0.528 | - |
| Any breastfeeding at birth or hospital discharge^b^ | 15,556/27,804 (55.95) | 12,243/18,696 (65.48) | **1.19 (1.17-1.21) P<0.001** | **1.2 (1.18-1.21) P<0.001** | **1.19 (1.17-1.21) P<0.001** | **1.15 (1.13-1.17) P<0.001** |
| Exclusive breastfeeding at 6-8 week review^c^ | 7,185/27,573 (26.06) | 6,793/19,247 (35.29) | **1.36 (1.32-1.40) P<0.001** | **1.38 (1.34-1.42) P<0.001** | **1.38 (1.34-1.43) P<0.001** | **1.31 (1.26-1.35) P<0.001** |
| Any breastfeeding at 6-8 week review^c^ | 9,883/27,573 (35.84) | 8,752/19,247 (45.47) | **1.28 (1.25-1.31) P<0.001** | **1.29 (1.26-1.32) P<0.001** | **1.30 (1.26-1.33) P<0.001** | **1.24 (1.20-1.27) P<0.001** |
| ***Perinatal outcomes^d^*** |  |  |  |  |  |  |
| Adverse perinatal outcome^e^†‡¥ | 1,282/28,065 (4.57) | 1,475/20,588 (7.16) | **1.52 (1.41-1.65) P<0.001** | **1.52 (1.41-1.65) P<0.001** | **1.58 (1.45-1.72) P<0.001** | **1.65 (1.50-1.81) P<0.001** |
| Intrapartum stillbirth or neonatal death | # (0.01) | 14/22,748 (0.06) | **10.27 (2.35-44.81) P=0.002** | NC | NC | NC |
| Admitted to a neonatal unit†‡¥ | 1,155/31,530 (3.66) | 1,043/22,243 (4.69) | **1.25 (1.15-1.37) P<0.001** | **1.26 (1.15-1.37) P<0.001** | **1.31 (1.19-1.44) P<0.001** | **1.37 (1.24-1.53) P<0.001** |
| Resuscitation requiring drugs and/or intubation†‡¥ | 78/28,399 (0.27) | 348/21,137 (1.65) | **5.3 (4.12-6.81) P<0.001** | **5.31 (4.12-6.85) P<0.001** | **5.73 (4.41-7.46) P<0.001** | **5.66 (4.32-7.42) P<0.001** |
| Apgar score <7 at 5 minutes†‡¥ | 110/31,630 (0.35) | 307/22,482 (1.37) | **3.93 (3.16-4.90) P<0.001** | **3.92 (3.14-4.89) P<0.001** | **3.75 (2.93-4.79) P<0.001** | **3.56 (2.76-4.60) P<0.001** |

1 Base model adjusted for year of delivery.

2 Model A adjusted for year of delivery and socio-demographic factors (maternal age, mother’s country of birth, marital status/registration type and socio-economic status).

3 Model B adjusted for variables in Model A and additionally adjusted for maternal medical and pregnancy-related factors (number of previous cesarean sections, any prior vaginal delivery, inter-pregnancy interval, maternal smoking status at booking, maternal BMI at booking, hypertensive disorder where † is shown, diabetes where ‡ is shown and pre-labor rupture of membranes where ¥ is shown).

4 Model C adjusted for variables in Model B and additionally adjusted for infant-related factors (sex of infant, gestational age at delivery and birth weight centile).

^a^ Women who died before discharge or were not discharged within 42 days of birth excluded from analysis of overnight readmission to hospital (n=2).

^b^ Intrapartum stillbirths (n=4) and births missing data on feeding at birth and hospital discharge (n=8,134, 14.9%) excluded from analysis of breastfeeding at birth or hospital discharge.

^c^ Intrapartum stillbirths (n=4), neonatal deaths (31) and births missing infant feeding data at 6-8wk review (n=7,783, 14.3%) excluded from analysis of breastfeeding outcomes at 6-8wks.

^d^ All perinatal outcomes exclude deaths due to congenital abnormalities (n=19) and any remaining intrapartum stillbirths (n=4) and births missing the outcome in question (n=842, 1.5% for admission to a neonatal unit; n=5,079, 9.3% for resuscitation; n=503, 0.9% for Apgar score) additionally excluded from analysis of neonatal unit admission, resuscitation and Apgar score.

^e^ Includes intrapartum stillbirth or neonatal death, admission to a neonatal unit, resuscitation requiring drugs and/or intubation or an Apgar score <7 at 5 minutes.

NC – not calculated because of low number of events.

# – numbers have not been shown to protect against potential disclosure risks

Bold text indicates statistically significant findings at the 5% level.
