# Supplementary material for: Planned mode of delivery after previous cesarean section and short-term maternal and perinatal outcomes: A population-based record linkage cohort study in Scotland
Source: PLoS Med. 2019 Sep 24;16(9):e1002913. doi: 10.1371/journal.pmed.1002913 (PMC6759152; doi:10.1371/journal.pmed.1002913)
Supplement: S6 Table — ERCS, elective repeat cesarean section; VBAC, vaginal birth after previous cesarean. (DOCX) [file pmed.1002913.s008.docx]

**S6 Table. Maternal and perinatal outcomes following planned VBAC with and without labor induction compared to ERCS at ≥ 39 weeks gestation**

|  | **ERCS** | **Planned VBAC without labor induction** | | | | |  | **Planned VBAC with labor induction** | | | | |
| --- | --- | --- | --- | --- | --- | --- | --- | --- | --- | --- | --- | --- |
|  | **n outcome events/total N (%)** | **n outcome events/total N (%)** | **Base model^1^ relative risk (95% CI)** | **Model A^2^ relative risk (95% CI)** | **Model B^3^**  **relative risk (95% CI)** | **Model C^4^ relative risk (95% CI)** |  | **n outcome events/total N (%)** | **Base model^1^ relative risk (95% CI)** | **Model A^2^ relative risk (95% CI)** | **Model B^3^**  **relative risk (95% CI)** | **Model C^4^ relative risk (95% CI)** |
| ***Maternal outcomes*** |  |  |  |  |  | - |  |  |  |  |  | - |
| Uterine rupture | 16/31,880 (0.05) | 44/18,340 (0.24) | **4.46**  **(2.45-8.12) P<0.001** | **4.47**  **(2.44-8.19) P<0.001** | **5.63**  **(2.73-11.63) P<0.001** | - |  | 13/4,338 (0.30) | **5.57**  **(2.66-11.65) P<0.001** | **5.77**  **(2.72-12.20) P<0.001** | **7.57**  **(3.24-17.68) P<0.001** | - |
| Peripartum hysterectomy | 9/31,880 (0.03) | #  (0.02) | 0.46  (0.12-1.75) P=0.255 | NC | NC | - |  | # | 0.64  (0.08-4.87)  P=0.667 | NC |  | - |
| Blood transfusion† | 149/31,880 (0.47) | 207/18,340 (1.13) | **2.14**  **(1.72-2.66) P<0.001** | **2.1**  **(1.69-2.61) P<0.001** | **2.29**  **(1.81-2.91) P<0.001** | - |  | 61/4,338 (1.41) | **2.66**  **(1.96-3.62) P<0.001** | **2.6**  **(1.91-3.53) P<0.001** | **2.86**  **(2.07-3.95) P<0.001** | - |
| Puerperal sepsis‡¥ | 48/31,880 (0.15) | 41/18,340 (0.22) | **1.74**  **(1.15-2.65) P=0.009** | **1.7**  **(1.12-2.58)**  **P=0.013** | **1.71**  **(1.06-2.75)**  **P=0.028** | - |  | 15/4,338 (0.35) | **2.66**  **(1.47-4.80) P=0.001** | **2.59**  **(1.44-4.66) P=0.002** | **2.46**  **(1.31-4.63) P=0.005** | - |
| Other puerperal infection‡¥ | 660/31,880 (2.07) | 421/18,340 (2.30) | 1.06  (0.93-1.20) P=0.393 | 1.05  (0.92-1.19) P=0.466 | **1.27**  **(1.10-1.46) P=0.001** | - |  | 119/4,338 (2.74) | **1.27**  **(1.03-1.55) P=0.022** | **1.25**  **(1.02-1.53) P=0.033** | **1.48**  **(1.20-1.84) P<0.001** | - |
| Surgical injury | 24/31,880 (0.08) | 34/18,340 (0.19) | **2.61**  **(1.56-4.39) P<0.001** | **2.64**  **(1.58-4.41) P<0.001** | NC | - |  | # | 1.62  (0.62-4.26) P=0.329 | 1.63  (0.62-4.30) P=0.323 | NC | - |
| Length of postnatal hospital stay >5 days†‡¥ | 729/31,880 (2.29) | 464/18,340 (2.53) | 0.94  (0.83-1.07) P=0.366 | 0.96  (0.84-1.08) P=0.484 | 1.04  (0.91-1.20) P=0.551 | - |  | 154/4,338 (3.55) | **1.33**  **(1.11-1.60) P=0.002** | **1.33**  **(1.11-1.60) P=0.002** | **1.38**  **(1.14-1.68)**  **P=0.001** | - |
| Readmission to hospital within 42 days of birth^a^†‡¥ | 822/31,879 (2.58) | 420/18,339 (2.29) | 0.91  (0.80-1.02)  P=0.116 | 0.91  (0.80-1.02)  P=0.111 | 0.99  (0.87-1.14)  P=0.901 | - |  | 131/4,338 (3.02) | 1.2  (1.00-1.46)  P=0.054 | 1.2  (0.99-1.45)  P=0.057 | **1.24**  **(1.02-1.52)**  **P=0.032** | - |
| Any breastfeeding at birth or hospital discharge^b^ | 15,556/27,804 (55.95) | 9,933/15,094 (65.81) | **1.2**  **(1.18-1.22) P<0.001** | **1.2**  **(1.18-1.21) P<0.001** | **1.19**  **(1.17-1.21) P<0.001** | **1.15**  **(1.13-1.17) P<0.001** |  | 2,262/3,538 (63.93) | **1.16**  **(1.13-1.19) P<0.001** | **1.2**  **(1.17-1.2) P<0.001** | **1.18**  **(1.14-1.22) P<0.001** | **1.11**  **(1.08-1.15) P<0.001** |
| Exclusive breastfeeding at 6-8 week review^c^ | 7,185/27,573 (26.06) | 5,605/15,479 (36.21) | **1.39**  **(1.35-1.43) P<0.001** | **1.4**  **(1.36-1.44) P<0.001** | **1.39**  **(1.35-1.44) P<0.001** | **1.32**  **(1.27-1.37) P<0.001** |  | 1,164/3,694 (31.51) | **1.21**  **(1.15-1.28) P<0.001** | **1.29**  **(1.23-1.36) P<0.001** | **1.33**  **(1.25-1.41) P<0.001** | **1.21**  **(1.14-1.29) P<0.001** |
| Any breastfeeding at 6-8 week review^c^ | 9,883/27,573 (35.84) | 7,165/15,479 (46.29) | **1.3**  **(1.27-1.34) P<0.001** | **1.3**  **(1.27-1.33) P<0.001** | **1.30**  **(1.26-1.33) P<0.001** | **1.24**  **(1.21-1.28) P<0.001** |  | 1,557/3,694 (42.15) | **1.19**  **(1.14-1.24) P<0.001** | **1.26**  **(1.21-1.31) P<0.001** | **1.28**  **(1.22-1.35) P<0.001** | **1.19**  **(1.13-1.25) P<0.001** |

**S6 Table continued**

|  | **ERCS** | **Planned VBAC without labor induction** | | | | |  | **Planned VBAC with labor induction** | | | | |
| --- | --- | --- | --- | --- | --- | --- | --- | --- | --- | --- | --- | --- |
|  | **n outcome events/total N (%)** | **n outcome events/total N (%)** | **Base model^1^ relative risk (95% CI)** | **Model A^2^ relative risk (95% CI)** | **Model B^3^**  **relative risk (95% CI)** | **Model C^4^ relative risk (95% CI)** |  | **n outcome events/total N (%)** | **Base model^1^ relative risk (95% CI)** | **Model A^2^ relative risk (95% CI)** | **Model B^3^**  **relative risk (95% CI)** | **Model C^4^ relative risk (95% CI)** |
| ***Perinatal outcomes^d^*** |  |  |  |  |  |  |  |  |  |  |  |  |
| Adverse perinatal outcome^e^†‡¥ | 1,282/28,065 (4.57) | 1,122/16,615 (6.75) | **1.42**  **(1.31-1.55) P<0.001** | **1.43**  **(1.32-1.56) P<0.001** | **1.50**  **(1.37-1.64) P<0.001** | **1.58**  **(1.43-1.74) P<0.001** |  | 346/3,925 (8.82) | **1.91**  **(1.69-2.16) P<0.001** | **1.89**  **(1.67-2.14) P<0.001** | **1.90**  **(1.67-2.17) P<0.001** | **2.07**  **(1.79-2.39) P<0.001** |
| Intrapartum stillbirth or neonatal death | #  (0.01) | #  (0.06) | **10.02**  **(2.21-45.43) P=0.003** | NC | NC | NC |  | #  (0.07) | **11.53**  **(2.02-65.99) P=0.006** | NC | NC | NC |
| Admitted to a neonatal unit†‡¥ | 1,155/31,530 (3.66) | 796/17,949 (4.43) | **1.18**  **(1.07-1.30) P=0.001** | **1.19**  **(1.08-1.31) P<0.001** | **1.25**  **(1.13-1.39) P<0.001** | **1.32**  **(1.18-1.47) P<0.001** |  | 242/4,242 (5.70) | **1.54**  **(1.33-1.78) P<0.001** | **1.53**  **(1.32-1.77) P<0.001** | **1.54**  **(1.32-1.79) P<0.001** | **1.67**  **(1.42-1.97) P<0.001** |
| Resuscitation requiring drugs and/or intubation†‡¥ | 78/28,399 (0.27) | 266/17,069 (1.56) | **4.98**  **(3.85-6.45) P<0.001** | **4.99**  **(3.85-6.47) P<0.001** | **5.44**  **(4.16-7.13) P<0.001** | **5.47**  **(4.15-7.20) P<0.001** |  | 79/4,011 (1.97) | **6.36**  **(4.62-8.75) P<0.001** | **6.35**  **(4.60-8.75) P<0.001** | **6.80**  **(4.86-9.50) P<0.001** | **6.89**  **(4.81-9.87) P<0.001** |
| Apgar score <7 at 5 minutes†‡¥ | 110/31,630 (0.35) | 232/18,108 (1.28) | **3.68**  **(2.93-4.63) P<0.001** | **3.69**  **(2.93-4.65) P<0.001** | **3.57**  **(2.77-5.61) P<0.001** | **3.45**  **(2.65-4.48) P<0.001** |  | 74/4,298 (1.72) | **4.97**  **(3.70-6.69) P<0.001** | **4.88**  **(3.62-6.58) P<0.001** | **4.56**  **(3.31-6.28) P<0.001** | **4.32**  **(3.07-6.09) P<0.001** |

1 Base model adjusted for year of delivery.

2 Model A adjusted for year of delivery and socio-demographic factors (maternal age, mother’s country of birth, marital status/registration type and socio-economic status).

3 Model B adjusted for variables in Model A and additionally adjusted for maternal medical and pregnancy-related factors (number of previous cesarean sections, any prior vaginal delivery, inter-pregnancy interval, maternal smoking status at booking, maternal BMI at booking, hypertensive disorder where † is shown, diabetes where ‡ is shown and pre-labor rupture of membranes where ¥ is shown).

4 Model C adjusted for variables in Model B and additionally adjusted for infant-related factors (sex of infant, gestational age at delivery and birth weight centile).

^a^ Women who died before discharge or were not discharged within 42 days of birth excluded from analysis of overnight readmission to hospital (n=2).

^b^ Intrapartum stillbirths (n=4) and births missing data on feeding at birth and hospital discharge (n=8,118, 14.9%) excluded from analysis of breastfeeding at birth or hospital discharge.

^c^ Intrapartum stillbirths (n=4), neonatal deaths (31) and births missing infant feeding data at 6-8wk review (n=7,777, 14.3%) excluded from analysis of breastfeeding outcomes at 6-8wks.

^d^ All perinatal outcomes exclude deaths due to congenital abnormalities (n=19) and any remaining intrapartum stillbirths (n=4) and births missing the outcome in question (n=814, 1.5% for admission to a neonatal unit; n=5,056, 9.3% for resuscitation; n=499, 0.9% for Apgar score) additionally excluded from analysis of neonatal unit admission, resuscitation and Apgar score.

^e^ Includes intrapartum stillbirth or neonatal death, admission to a neonatal unit, resuscitation requiring drugs and/or intubation or an Apgar score <7 at 5 minutes.

NC – not calculated because of low number of events.

# – numbers or numbers and percentages have not been shown to protect against potential disclosure risks

Bold text indicates statistically significant findings at the 5% level.
