# Supplementary material for: Planned mode of delivery after previous cesarean section and short-term maternal and perinatal outcomes: A population-based record linkage cohort study in Scotland
Source: PLoS Med. 2019 Sep 24;16(9):e1002913. doi: 10.1371/journal.pmed.1002913 (PMC6759152; doi:10.1371/journal.pmed.1002913)
Supplement: S7 Table — ERCS, elective repeat cesarean section; VBAC, vaginal birth after previous cesarean. (DOCX) [file pmed.1002913.s009.docx]

**S7 Table. Maternal and perinatal outcomes following successful VBAC and in-labor non-elective repeat cesarean section compared to ERCS at ≥ 39 weeks gestation**

|  | **ERCS** | **Successful VBAC** | | | | |  | **In-labor non-elective repeat cesarean section** | | | | |
| --- | --- | --- | --- | --- | --- | --- | --- | --- | --- | --- | --- | --- |
|  | **n outcome events/total N (%)** | **n outcome events/total N (%)** | **Base model^1^ relative risk (95% CI)** | **Model A^2^ relative risk (95% CI)** | **Model B^3^**  **relative risk (95% CI)** | **Model C^4^ relative risk (95% CI)** |  | **n outcome events/total N (%)** | **Base model^1^ relative risk (95% CI)** | **Model A^2^ relative risk (95% CI)** | **Model B^3^**  **relative risk (95% CI)** | **Model C^4^ relative risk (95% CI)** |
| ***Maternal outcomes*** |  |  |  |  |  |  |  |  |  |  |  |  |
| Uterine rupture | 16/31,880 (0.05) | 8/16,441 (0.05) | 0.91  (0.39-2.13) P=0.825 | 0.92  (0.39-2.17) P=0.854 | 0.95  (0.39-2.34) P=0.916 | - |  | 49/6,317 (0.78) | **14.52**  **(8.02-26.28) P<0.001** | **14.66**  **(8.01-26.84) P<0.001** | **15.36**  **(7.94-29.69) P<0.001** | - |
| Peripartum hysterectomy | 9/31,880 (0.03) | #  (0.02) | 0.52  (0.14-1.92) P=0.325 | NC | NC | - |  | # | 0.44  (0.06-3.44) P=0.433 | NC | NC | - |
| Blood transfusion† | 149/31,880 (0.47) | 180/16,441 (1.09) | **2.08**  **(1.66-2.60) P<0.001** | **2.04**  **(1.63-2.56) P<0.001** | **2.29**  **(1.77-2.96) P<0.001** | - |  | 88/6,317 (1.39) | **2.63**  **(2.00-3.45) P<0.001** | **2.56**  **(1.95-3.36) P<0.001** | **2.58**  **(1.96-3.41) P<0.001** | - |
| Puerperal sepsis‡¥ | 48/31,880 (0.15) | 31/16,441 (0.19) | 1.47  (0.94-2.31) P=0.094 | 1.44  (0.92-2.25) P=0.116 | 1.47  (0.87-2.46) P=0.147 | - |  | 26/6,317 (0.41) | **3.23**  **(1.97-5.28) P<0.001** | **3.16**  **(1.95-5.13) P<0.001** | **2.81**  **(1.67-4.71) P<0.001** | - |
| Other puerperal infection‡¥ | 660/31,880 (2.07) | 258/16,441 (1.57) | **0.72**  **(0.62-0.83) P<0.001** | **0.71**  **(0.61-0.82) P<0.001** | 0.87  (0.74-1.03) P=0.105 | - |  | 282/6,317 (4.46) | **2.1**  **(1.81-2.43) P<0.001** | **2.1**  **(1.81-2.44) P<0.001** | **2.19**  **(1.88-2.56) P<0.001** | - |
| Surgical injury | 24/31,880 (0.08) | #  (0.01) | **0.17**  **(0.04-0.71) P=0.015** | **0.17**  **(0.04-0.72) P=0.016** | NC | - |  | 37/6,317  (0.59) | **8.31**  **(4.97-13.91) P<0.001** | **8.56**  **(5.18-14.15) P<0.001** | NC | - |
| Length of postnatal hospital stay >5 days†‡¥ | 729/31,880 (2.29) | 294/16,441 (1.79) | **0.66**  **(0.57-0.77) P<0.001** | **0.67**  **(0.58-0.77) P<0.001** | **0.69**  **(0.59-0.81) P<0.001** | - |  | 326/6,317 (5.16) | **1.97**  **(1.71-2.26) P<0.001** | **2.04**  **(1.77-2.34) P<0.001** | **2.06**  **(1.78-2.38) P<0.001** | - |
| Readmission to hospital within 42 days of birth^a^†‡¥ | 822/31,879 (2.58) | 380/16,440 (2.31) | 0.92  (0.81-1.04) P=0.173 | 0.91  (0.80-1.04) P=0.157 | 1.00  (0.87-1.16) P=0.973 | - |  | 174/6,317 (2.75) | 1.10  (0.93-1.30) P=0.270 | 1.1  (0.93-1.30) P=0.258 | 1.13  (0.95-1.35)  P=0.179 | - |
| Any breastfeeding at birth or hospital discharge^b^ | 15,556/27,804 (55.95) | 8,619/13,374 (64.45) | **1.17**  **(1.15-1.19) P<0.001** | **1.19**  **(1.17-1.21) P<0.001** | **1.20**  **(1.18-1.22) P<0.001** | **1.16**  **(1.14-1.18) P<0.001** |  | 3,624/5,322 (68.09) | **1.24**  **(1.21-1.27) P<0.001** | **1.22**  **(1.19-1.24) P<0.001** | **1.17**  **(1.14-1.20) P<0.001** | **1.12**  **(1.09-1.15) P<0.001** |
| Exclusive breastfeeding at 6-8 week review^c^ | 7,185/27,573 (26.06) | 4,827/13,752 (35.10) | **1.35**  **(1.30-1.39) P<0.001** | **1.4**  **(1.35-1.44) P<0.001** | **1.39**  **(1.34-1.44) P<0.001** | **1.32**  **(1.27-1.36) P<0.001** |  | 1,966/5,495 (35.78) | **1.37**  **(1.32-1.43) P<0.001** | **1.34**  **(1.29-1.39) P<0.001** | **1.36**  **(1.30-1.43) P<0.001** | **1.27**  **(1.21-1.34) P<0.001** |
| Any breastfeeding at 6-8 week review^c^ | 9,883/27,573 (35.84) | 6,153/13,752 (44.74) | **1.26**  **(1.23-1.29) P<0.001** | **1.29**  **(1.26-1.32) P<0.001** | **1.30**  **(1.27-1.34) P<0.001** | **1.25**  **(1.21-1.28) P<0.001** |  | 2,599/5,495 (47.30) | **1.33**  **(1.29-1.38) P<0.001** | **1.29**  **(1.25-1.33) P<0.001** | **1.28**  **(1.23-1.33) P<0.001** | **1.21**  **(1.16-1.26) P<0.001** |

**S7 Table continued**

|  | **ERCS** | **Successful VBAC** | | | | |  | **In-labor non-elective repeat cesarean section** | | | | |
| --- | --- | --- | --- | --- | --- | --- | --- | --- | --- | --- | --- | --- |
|  | **n outcome events/total N (%)** | **n outcome events/total N (%)** | **Base model^1^ relative risk (95% CI)** | **Model A^2^ relative risk (95% CI)** | **Model B^3^**  **relative risk (95% CI)** | **Model C^4^ relative risk (95% CI)** |  | **n outcome events/total N (%)** | **Base model^1^ relative risk (95% CI)** | **Model A^2^ relative risk (95% CI)** | **Model B^3^**  **relative risk (95% CI)** | **Model C^4^ relative risk (95% CI)** |
| ***Perinatal outcomes^d^*** |  |  |  |  |  |  |  |  |  |  |  |  |
| Adverse perinatal outcome^e^†‡¥ | 1,282/28,065 (4.57) | 957/14,898 (6.42) | **1.36**  **(1.24-1.48) P<0.001** | **1.35**  **(1.23-1.47) P<0.001** | **1.40**  **(1.27-1.54) P<0.001** | **1.48**  **(1.33-1.64) P<0.001** |  | 518/5,690 (9.10) | **1.97**  **(1.77-2.19) P<0.001** | **2.02**  **(1.81-2.25) P<0.001** | **1.98**  **(1.76-2.21) P<0.001** | **2.06**  **(1.83-2.32) P<0.001** |
| Intrapartum stillbirth or neonatal death | #  (0.01) | 8/16,434 (0.05) | **8.12**  **(1.74-37.93) P=0.008** | NC | NC | NC |  | 6/6,314  (0.10) | **15.9**  **(3.23-78.25) P<0.001** | NC | NC | NC |
| Admitted to a neonatal unit†‡¥ | 1,155/31,530 (3.66) | 659/16,145 (4.08) | 1.08  (0.98-1.20) P=0.110 | 1.08  (0.98-1.20) P=0.128 | **1.13**  **(1.01-1.26) P=0.032** | **1.20**  **(1.07-1.35) P=0.003** |  | 384/6,098 (6.30) | **1.71**  **(1.52-1.93) P<0.001** | **1.76**  **(1.56-1.98) P<0.001** | **1.72**  **(1.52-1.95) P<0.001** | **1.80**  **(1.58-2.06) P<0.001** |
| Resuscitation requiring drugs and/or intubation†‡¥ | 78/28,399 (0.27) | 239/15,237 (1.57) | **5.06**  **(3.90-6.58) P<0.001** | **5.04**  **(3.87-6.56) P<0.001** | **5.55**  **(4.20-7.33) P<0.001** | **5.50**  **(4.15-7.30) P<0.001** |  | 109/5,900 (1.85) | **5.9**  **(4.39-7.93) P<0.001** | **6.05**  **(4.48-8.15) P<0.001** | **6.07**  **(4.49-8.21) P<0.001** | **5.98**  **(4.35-8.23) P<0.001** |
| Apgar score <7 at 5 minutes†‡¥ | 110/31,630 (0.35) | 181/16,228 (1.12) | **3.21**  **(2.53-4.07) P<0.001** | **3.17**  **(2.50-4.03) P<0.001** | **2.90**  **(2.22-3.78) P<0.001** | **2.81**  **(2.14-3.70) P<0.001** |  | 126/6,254 (2.01) | **5.84**  **(4.51-7.57) P<0.001** | **6.00**  **(4.61-7.80) P<0.001** | **5.61**  **(4.26-7.38) P<0.001** | **5.38**  **(4.02-7.21) P<0.001** |

1 Base model adjusted for year of delivery.

2 Model A adjusted for year of delivery and socio-demographic factors (maternal age, mother’s country of birth, marital status/registration type and socio-economic status).

3 Model B adjusted for variables in Model A and additionally adjusted for maternal medical and pregnancy-related factors (number of previous cesarean sections, any prior vaginal delivery, inter-pregnancy interval, maternal smoking status at booking, maternal BMI at booking, hypertensive disorder where † is shown, diabetes where ‡ is shown and pre-labor rupture of membranes where ¥ is shown).

4 Model C adjusted for variables in Model b and additionally adjusted for infant-related factors (sex of infant, gestational age at delivery and birth weight centile).

^a^ Women who died before discharge or were not discharged within 42 days of birth excluded from analysis of overnight readmission to hospital (n=2).

^b^ Intrapartum stillbirths (n=4) and births missing data on feeding at birth and hospital discharge (n=8,134, 14.9%) excluded from analysis of breastfeeding at birth or hospital discharge.

^c^ Intrapartum stillbirths (n=4), neonatal deaths (31) and births missing infant feeding data at 6-8wk review (n=7,783, 14.3%) excluded from analysis of breastfeeding outcomes at 6-8wks.

^d^ All perinatal outcomes exclude deaths due to congenital abnormalities (n=19) and any remaining intrapartum stillbirths (n=4) and births missing the outcome in question (n=842, 1.5% for admission to a neonatal unit; n=5,079, 9.3% for resuscitation; n=503, 0.9% for Apgar score) additionally excluded from analysis of neonatal unit admission, resuscitation and Apgar score.

^e^ Includes intrapartum stillbirth or neonatal death, admission to a neonatal unit, resuscitation requiring drugs and/or intubation or an Apgar score <7 at 5 minutes.

NC – not calculated because of low number of events.

# – numbers or numbers and percentages have not been shown to protect against potential disclosure risks

Bold text indicates statistically significant findings at the 5% level.
