# Supplementary material for: Planned mode of delivery after previous cesarean section and short-term maternal and perinatal outcomes: A population-based record linkage cohort study in Scotland
Source: PLoS Med. 2019 Sep 24;16(9):e1002913. doi: 10.1371/journal.pmed.1002913 (PMC6759152; doi:10.1371/journal.pmed.1002913)
Supplement: S8 Table — SSBID, Scottish Stillbirth and Infant Death Survey. (DOCX) [file pmed.1002913.s010.docx]

**S8 Table. SSBID identified intrapartum stillbirth or neonatal death excluding deaths from congenital abnormalities by planned and actual mode of delivery after previous cesarean section**

| **Exposure** | **n outcome events/total n (%)** | **Base model^1^ relative risk (95% CI)** |
| --- | --- | --- |
| ERCS | # (0.01) | 1 |
| Planned VBAC | 12/18,958 (0.06) | **8.16 (1.82-36.60)**  **P=0.006** |
|  |  |  |
| ERCS | # (0.01) | 1 |
| Planned VBAC without labor induction | # (0.06) | **7.45 (1.60-34.58) P=0.010** |
| Planned VBAC with labor induction | # (0.09) | **11.75 (1.95-70.92) P=0.007** |
|  |  |  |
| ERCS | # (0.01) | 1 |
| Successful VBAC | 7/13,593 (0.05) | **6.63 (1.37-32.02) P=0.018** |
| In-labor non-elective repeat cesarean section | 5/5,365 (0.09) | **12.13 (2.34-62.92) P=0.003** |

1 Base model adjusted for year of delivery.

# numbers have not been shown to protect against potential disclosure risks.

Bold text indicates statistically significant findings at the 5% level.
